# Identification of transcriptional regulatory elements for *Ntng1* and *Ntng2* genes in mice

**DOI:** 10.1186/1756-6606-7-19

**Published:** 2014-03-19

**Authors:** Kunio Yaguchi, Sachiko Nishimura-Akiyoshi, Satoshi Kuroki, Takashi Onodera, Shigeyoshi Itohara

**Affiliations:** 1 RIKEN Brain Science Institute, 2-1 Hirosawa, Wako, Saitama, Japan; 2 Department of Molecular Immunology, University of Tokyo, 1-1-1 Yayoi, Bunkyo, Tokyo, Japan

## Abstract

**Background:**

Higher brain function is supported by the precise temporal and spatial regulation of thousands of genes. The mechanisms that underlie transcriptional regulation in the brain, however, remain unclear. The *Ntng1* and *Ntng2* genes, encoding axonal membrane adhesion proteins netrin-G1 and netrin-G2, respectively, are paralogs that have evolved in vertebrates and are expressed in distinct neuronal subsets in a complementary manner. The characteristic expression patterns of these genes provide a part of the foundation of the cortical layer structure in mammals.

**Results:**

We used gene-targeting techniques, bacterial artificial chromosome (BAC)-aided transgenesis techniques, and *in vivo* enhancer assays to examine transcriptional mechanisms *in vivo* to gain insight into how the characteristic expression patterns of these genes are acquired. Analysis of the gene expression patterns in the presence or absence of netrin-G1 and netrin-G2 functional proteins allowed us to exclude the possibility that a feedback or feedforward mechanism mediates their characteristic expression patterns. Findings from the BAC deletion series revealed that widely distributed combinations of *cis*-regulatory elements determine the differential gene expression patterns of these genes and that major *cis*-regulatory elements are located in the 85–45 kb upstream region of *Ntng2* and in the 75–60 kb upstream region and intronic region of *Ntng1. In vivo* enhancer assays using 2-kb evolutionarily conserved regions detected enhancer activity in the distal upstream regions of both genes.

**Conclusions:**

The complementary expression patterns of *Ntng1* and *Ntng2* are determined by transcriptional *cis*-regulatory elements widely scattered in these loci. The *cis*-regulatory elements characterized in this study will facilitate the development of novel genetic tools for functionally dissecting neural circuits to better understand vertebrate brain function.

## Background

Development and function of neural circuits in the vertebrate brain are supported by the orchestration of the spatial and temporal expression of genes in the brain. Disturbances in the transcriptional mechanisms are associated with major neurologic disorders, including mental deficiency, cerebral palsy, epilepsy, schizophrenia, and autism [1,2]. The characterization of the *cis*-acting regulatory sequences supporting proper gene expression, however, is insufficient. Unlike coding-sequences, distant *cis*-acting transcription regulatory sequences involved in particular biologic processes are difficult to identify because they locate in the vast and poorly characterized non-coding portion of the genome and may be located hundreds or thousands of kilobase (kb) pairs away from the target genes they regulate [3]. Therefore, the identification of sequences that control the location and timing of gene expression in the brain is a crucial challenge in neuroscience.

Netrin-G1 (*Ntng1*) and netrin-G2 (*Ntng2)*, also called laminet 1 and laminet 2, respectively, are UNC-6/netrin family members [4-6]. Classic netrins are phylogenetically conserved diffusible chemoattractants of axon guidance molecules [7-9]. Unlike classic netrins, netrin-Gs are linked to the plasma membrane surface by a glycosyl-phosphatidylinositol linkage, have no invertebrate orthologs, and lack affinity to the known netrin receptor families [4,5]. Netrin-G1 and netrin-G2 interact with specific receptors. Netrin-G1 interacts with netrin-G1 ligand (NGL1) [10], whereas netrin-G2 interacts with NGL2 [11,12]. *Ntng1* and *Ntng2* are clearly expressed in distinct neuronal subsets in a complementary manner [5,6] and have different roles in distinct neuronal circuits [12,13]. The differential expression patterns are highly conserved in primates such as marmosets and macaque [14], and also likely in humans [15]. Human genetics studies have detected single nucleotide polymorphisms in both *NTNG1* and *NTNG2* in association with schizophrenia [16-18] and rearrangements in *NTNG1* in a patient with Rett syndrome [19,20]. *NTNG1* and *NTNG2* alterations might also be involved in bipolar disease [21,22]. Studies with netrin-G1 and netrin-G2 knock-out (KO) mice have revealed the crucial significance of their differential expression in higher brain functions [12]. Elucidation of the transcriptional mechanisms that regulate the complementary expression of *Ntng1* and *Ntng2* will help to clarify the mechanisms of vertebrate-specific neural circuit formation and function, and will act as a springboard for novel cutting-edge research designed to gain a better understanding of the basis of higher brain function in vertebrates.

Mouse *Ntng1* and *Ntng2* are large genes that span more than 362 kb and 53 kb, respectively. Their *cis*-regulatory elements may also be widely distributed. The insert size limitation of high-copy plasmid constructs makes the use of these plasmids impractical for identifying more distant *cis*-regulatory elements. Bacterial artificial chromosomes (BACs), however, allow for the modification of genomic DNA, including large areas (~200 kb) that can cover the whole gene locus [23,24]. BAC DNA recombination methods have also been improved [25]. Moreover, recent progress in genome research allows for the identification of Evolutionarily Conserved Regions (ECRs) among species throughout their genomes. ECRs are useful for predicting functional domains without prior knowledge of the actual function [26-28]. Taking advantage of these tools, we constructed BAC transgenic mice to analyze the molecular mechanisms that underlie the regulation of *Ntng1* and *Ntng2* transcription.

In the present study, we analyzed the transcriptional mechanisms that regulate the characteristic expression patterns of *Ntng1* and *Ntng2*. First, we examined the transcriptional properties of *Ntng1* and *Ntng2* based on the expression of the *Escherichia coli beta*-galactosidase gene (*LacZ*) in knock-in (KI) mice. Second, to examine possible *trans-*mechanisms for the differential expression in *Ntng1* and *Ntng2* genes, we investigated *LacZ* reporter expression in the presence and absence of netrin-G1 or netrin-G2. Third, to identify the *cis*-regulatory elements of *Ntng1* and *Ntng2*, we performed expression analyses of both genes using a BAC transgenic mouse technique and transgenic enhancer assays with a heterologous minimal promoter in mice. Our findings revealed that *cis*-elements that are widely and distantly distributed from the transcription start site (TSS) determine the unique expression patterns of these genes.

## Results

### *LacZ*-KI mice exhibit complementary expression patterns of *Ntng1* and *Ntng2*

**Figure 1:**
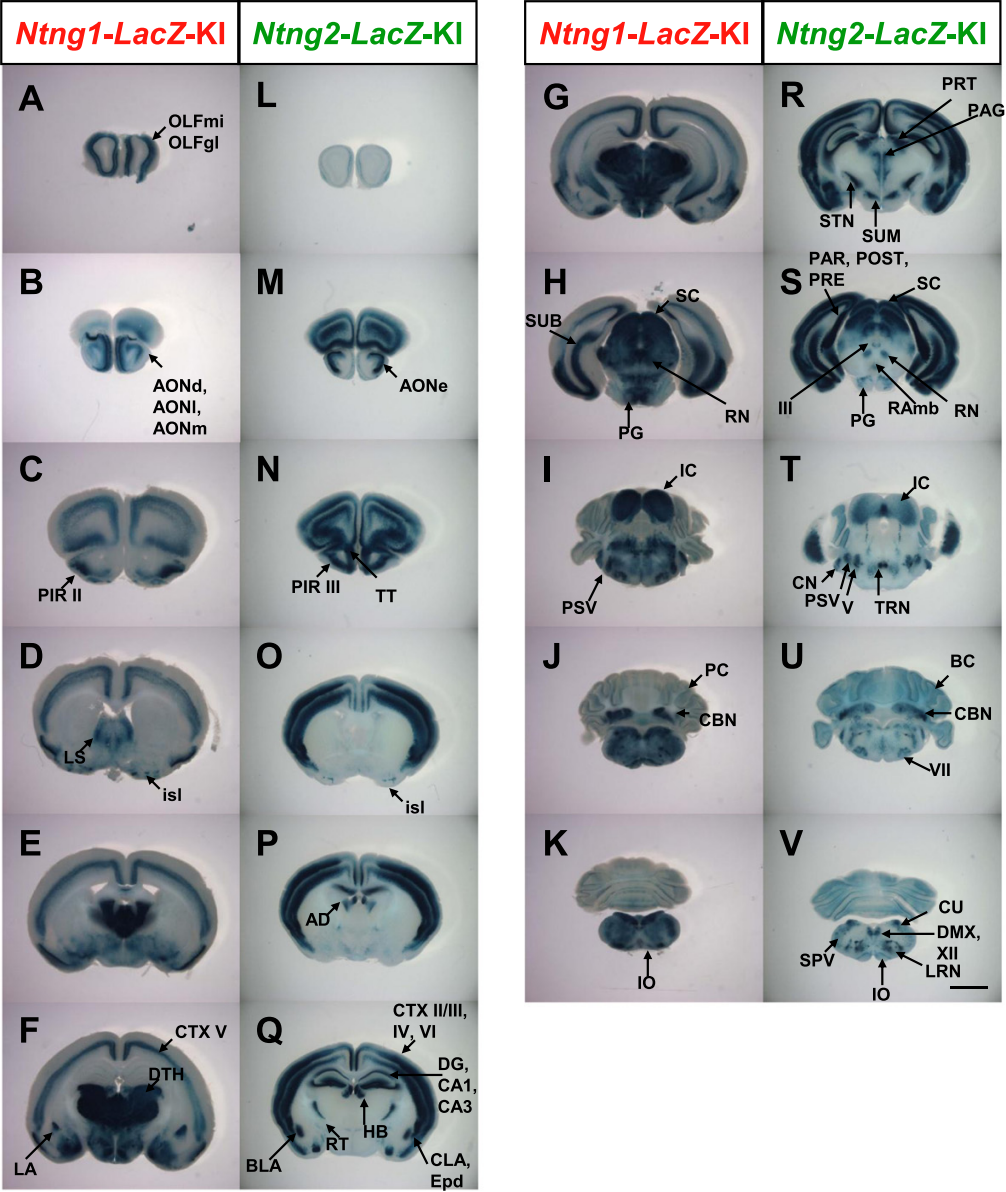
**X-gal staining of *Ntng1*- and *Ntng2*-*LacZ*-KI mouse brains. (A–K)** X-gal staining of *Ntng1*-*LacZ*-KI mouse brains. **(L–V)** X-gal staining of *Ntng2*-*LacZ*-KI mouse brains. Coronal slices (400 μm) at P21 were stained with X-gal staining solution. In panels A–K and L–V, the stained sections are arranged from anterior to posterior. AD, anterodorsal nucleus of the thalamus; AON, anterior olfactory nucleus; BC, Bergmann glia cells; BLA, basolateral amygdala nucleus; CA1, cornu ammonis 1 field of the hippocampus; CA3, cornu ammonis 3 field of the hippocampus; CBN, cerebellar nuclei; CLA, claustrum; CN, cochlear nuclei; CTX, cerebral cortex; CU, cuneate nucleus; DG, dentate gyrus; DMX, dorsal motor nucleus of the vagus nerve; DTH, dorsal thalamus; ENT, entorhinal area; Epd, endopiriform nucleus, dorsal part; HB, habenula; IC, inferior colliculus; isl, olfactory tubercle; Islands of Calleja; IO, inferior olivary complex; LA, lateral amygdala nucleus; LRN, lateral reticular nucleus*;* LS, lateral septal nucleus; OLFgl, olfactory bulb, glomerular layer; OLFmi, olfactory bulb, mitral layer; PAG, periaqueductal gray, dorsal division; PC, Purkinje cell; PIR, piriform area; PG, pontine gray; PRT, pretectal region; PSV, principal sensory nucleus of the trigeminal; RAmb, midbrain raphe nuclei; RN, red nucleus; RT, reticular nucleus of the thalamus; SC, superior colliculus; SPV, spinal nucleus of the trigeminal; STN, subthalamic nucleus; SUB, subiculum; SUM, supramammillary nucleus; TRN, tegmental reticular nucleus; TT, taenia tecta; III, oculomotor nucleus; V, trigeminal motor nucleus; VII, facial motor nucleus; XII, hypoglossal nucleus. Scale bar: 2.0 mm for all panels.

To investigate the spatial distribution of *Ntng1* and *Ntng2* expression in detail, we generated *Ntng1- and Ntng2-LacZ-KI* mice. These mice carry *LacZ* fused with a nuclear localization signal and an SV40 polyadenylation signal (NLS-*LacZ*-pA) in-frame in exon 2 of the *Ntng1* or *Ntng2* gene, and thus the transcriptional activities of these genes can be monitored by examining *LacZ* activity at a single cell resolution. We performed 5-bromo-4-chloro-3-indolyl-β-d-galactoside (X-gal) staining of *Ntng1*- and *Ntng2-LacZ*-KI mouse brains at postnatal day 21 (P21). The expression patterns of these genes are largely maintained throughout life. The findings from the *Ntng1-LacZ*-KI mice and *Ntng2-LacZ*-KI mice are summarized in Additional file 1: Table S1, and representative photos are shown in Figure 1 and Additional file 2: Figure S1.

In the cerebral cortex, *Ntng1* was expressed in neurons in layer V (Figure 1C to G, Additional file 2: Figure S1C), whereas *Ntng2* was expressed in neurons in layers II/III, IV, and VI (Figure 1N to S, Additional file 2: Figure S1H). The claustrum and endopiriform nucleus are closely associated with the cortex. Consistent with data from a previous study in monkeys and rats [14], only *Ntng2* was expressed in the claustrum and endopiriform nucleus (Figure 1O to Q). In the olfactory areas, *Ntng1* was highly expressed in the olfactory bulb mitral cells and glomerular cells (Figure 1A). Only *Ntng2* was expressed in the taenia tecta (Figure 1N). In the anterior olfactory nucleus, *Ntng1* was expressed in the neurons of dorsal, lateral, and medial parts (Figure 1B, Additional file 2: Figure S1A), whereas *Ntng2* was expressed in the neurons of the external part (Figure 1M, Additional file 2: Figure S1F). In the piriform cortex, *Ntng1* was expressed in layer II neurons (Figure 1C to G, Additional file 2: Figure S1B), and *Ntng2* was expressed in layer III neurons (Figure 1 N to R, Additional file 2: Figure S1G). *Ntng2* was expressed in dentate granule cells and hippocampal pyramidal neurons (Figure 1Q and R). *Ntng1,* but not *Ntng2,* was strongly expressed in the subiculum (Figure 1H and S). Conversely, *Ntng2,* but not *Ntng1,* was strongly expressed in the parasubiculum, postsubiculum, and presubiculum (Figure 1H and S). In the entorhinal area, *Ntng1* was expressed in layer II of the lateral entorhinal area and layer III throughout the entorhinal area (Additional file 2: Figure S1D), and *Ntng2* was expressed at a relatively low level in layer II of the medial entorhinal area and layer III of the lateral entorhinal area (Additional file 2: Figure S1I). In the amygdala, *Ntng1* was expressed in the lateral amygdala nucleus (Figure 1F, Additional file 2: Figure S1E), whereas *Ntng2* was expressed in the basolateral amygdala nucleus (Figure 1Q, Additional file 2: Figure S1J). In the striatum, *Ntng1* and *Ntng2* were expressed in the olfactory tubercles and Islands of Calleja (Figure 1D and O). *Ntng1* was modestly expressed in the lateral and medial septal nuclei (Figure 1D). In the cerebellum, *Ntng1* and *Ntng2* were strongly expressed in the cerebellar nuclei (Figure 1J and U). *Ntng2* was modestly expressed in the Bergmann glia (Figure 1U). *Ntng1* was weakly expressed in the Purkinje cells (Figure 1J). In the thalamus and peri-thalamic region, *Ntng1* was expressed in most nuclei except for the reticular thalamic nucleus and the habenula (Figure 1F), whereas complementary *Ntng2* expression was observed in the reticular thalamic nucleus, medial and lateral habenula, and anterodorsal nucleus of the thalamus (Figure 1P and Q). In the hypothalamus, *Ntng1* was expressed in the medial mammillary nucleus, whereas *Ntng2* was highly expressed in the supramammillary nucleus and subthalamic nucleus (Figure 1R). In the midbrain, although both genes were expressed in the superior and inferior colliculi (Figure 1H, I, S, and T), the expression gradients differed in distinct layers of these structures. The red nucleus expressed both genes (Figure 1H and S). *Ntng2* expression was detected in the dorsal division of the periaqueductal gray, pretectal region, oculomotor nucleus, and midbrain raphe nuclei (Figure 1R and S). In the pons, both genes were expressed in the pontine gray and principal sensory nucleus of the trigeminal nerve (Figure 1H, I, S, and T). *Ntng2* was further expressed in the tegmental reticular nucleus, trigeminal motor nucleus, and facial motor nucleus (Figure 1T). In the medulla, both genes were expressed in the inferior olivary complex (Figure 1K and V). *Ntng2* was modestly expressed in the cochlear nuclei, cuneate nucleus, spinal trigeminal nucleus, dorsal motor nucleus of the vagus nerve, lateral reticular nucleus, and hypoglossal nucleus (Figure 1U and V).

Consistent with previous *in situ* hybridization findings [4-6], X-gal staining for *Ntng1- and Ntng2-LacZ-KI* mice revealed differential expression patterns of *Ntng1* and *Ntng2* in most brain regions.

### Examination of gene regulatory mechanisms between *Ntng1* and *Ntng2* acting *in trans*

**Figure 2:**
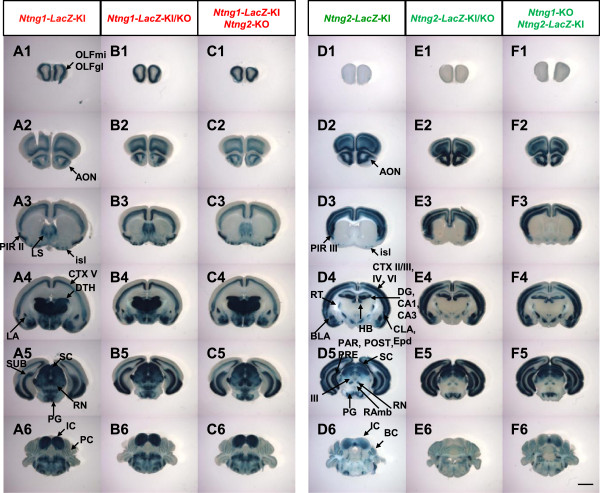
***LacZ *staining in the brains of *Ntng1*- and *Ntng2*- KI/KO mice. (A)***LacZ* staining of *Ntng1*^(*LacZ*/+)^ mouse brain. **(B)***LacZ* staining of *Ntng1*^(*LacZ/GFP*)^ mouse brain. This mouse lacked netrin-G1. **(C)***LacZ* staining of *Ntng1*^(*LacZ*/+)^ and *Ntng2*^(−/−)^ mouse brain. **(D)***LacZ* staining of *Ntng2*^(*LacZ*/+)^ mouse brain. **(E)***LacZ* staining of *Ntng2*^(*LacZ*/-)^ mouse brain. **(F)***LacZ* staining of *Ntng1*^(*GFP/GFP*)^ and *Ntng2*^(*LacZ*/+)^ mouse brain. Coronal slices (400 μm) at P21 were stained with X-gal solution. The panels are arranged from anterior (Top) to posterior (Bottom). Scale bar: 2.0 mm for all panels.

We hypothesized that the complementary expression of *Ntng1* and *Ntng2* is regulated by *trans-*acting mechanisms in a manner similar to the expression of olfactory receptor (OR) [29], immunoglobulin, and T-cell receptor genes [30,31]. In allelic exclusion, in the immune system, a functional V(D)J rearrangement in one allele suppresses rearrangements in another allele by negative feedback regulation. One particular OR gene is activated by a *cis-*acting locus control region (H region), and functional OR molecules transmit inhibitory signals to block further activation of additional OR genes. For example, functional expression of netrin-G1 protein might inhibit *Ntng2* transcription, and that of netrin-G2 protein might inhibit *Ntng1* transcription. Alternatively, they may also accelerate the expression of their own genes. We tested this possibility by examining expression patterns in the *LacZ* KI allele in the absence of netrin-G1 or netrin-G2. We interbred *Ntng-LacZ*-KI mice and other *Ntng1*-KO or *Ntng2-*KO variants and investigated the expression patterns of the *LacZ* gene in the presence or absence of netrin-G1 or netrin-G2. We observed no changes in *LacZ* expression in the *Ntng1-* and *Ntng2-LacZ*-KI alleles under these conditions (Figure 2). These results clearly ruled out the possibility that *trans* mechanisms underlie the differential expression of *Ntng1* and *Ntng2*.

### *Cis*-regulatory elements responsible for endogenous *Ntng2* expression

**Figure 3:**
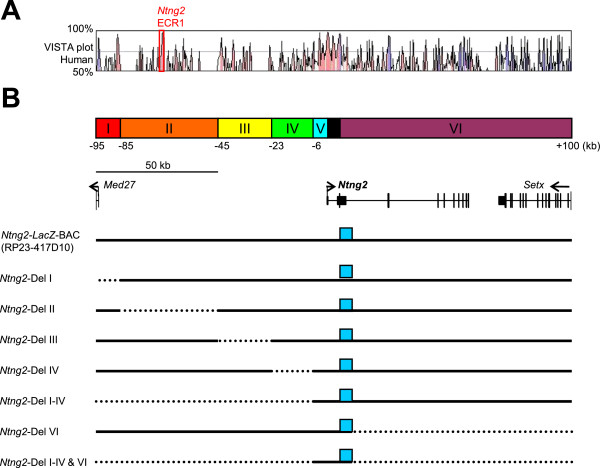
**Highly conserved sequences in *Ntng2 *loci and strategy for analyzing *cis*-regulatory elements in transgenic mice. (A)** Comparative genomic analysis of the mouse *Ntng2*-BAC sequence (mouse Dec. 2011 [GRCm38/mm10] assembly; chr2: 29,153,280-29,347,847) by the VISTA genome browser [ http://genome.lbl.gov/vista/index.shtml] [32]. Percent nucleotide identities between mouse and human DNA sequences were plotted as a function of the position along the mouse sequence. Peaks of evolutionary conserved exons of *Ntng2* and neighboring genes are shaded blue. Aligned regions with more than 70% identity over 100 bases are shaded pink. **(B)** Schematic diagrams of the *Ntng2* deletion series. The *Ntng2*-BAC sequences are divided into 6 segments (I–VI indicated in colored boxes) along with ECR distribution. Genomic organizations of the mouse *Ntng2* and neighbor gene loci are indicated. Short vertical lines indicate positions of exons and arrowheads indicate transcriptional directions. Broken and solid lines represent deleted and preserved regions in the BAC constructs, respectively. Blue boxes on the lines represent the reporter *LacZ* cassette inserted in each BAC construct. Note that the reporter *LacZ* cassette, ECRs, and minimal promoter sequences are not represented in accurate scales.

To systematically examine the transcriptional mechanisms underlying the characteristic expression patterns of *Ntng2* in young adult mouse brains, the *cis*-regulatory elements of *Ntng2* were analyzed in BAC transgenic mice. The *Ntng2*-BAC clone (RP23-417D10) that covered the 195-kb region of the locus between −95 kb and +100 kb of the *Ntng2* TSS was used to generate *Ntng2-LacZ*-BAC transgenic mice (Figure 3). The adjacent genes, *Med27* and *Setx*, locate at 94 kb upstream and 70 kb downstream of the *Ntng2* TSS, respectively.

Using the Red/ET recombination technique [32], the *Ntng2-*BAC clone was inserted with an NLS-*LacZ*-pA cassette at the translation start site in exon 2. The resulting *Ntng2-LacZ-*BAC construct was microinjected into the pronuclei of fertilized mouse eggs to establish transgenic founders. Transgenic founder mice were transcardially perfused with 4% paraformaldehyde at P21. Fixed mouse brains were sliced at a thickness of 400 μm and stained for *LacZ* activity using X-gal. Only founders that carried the end sequences of the transgene were selected and analyzed. Throughout this study, the same protocol for detecting *LacZ* expression was used to analyze expression patterns in transgenic mice.

**Figure 4:**
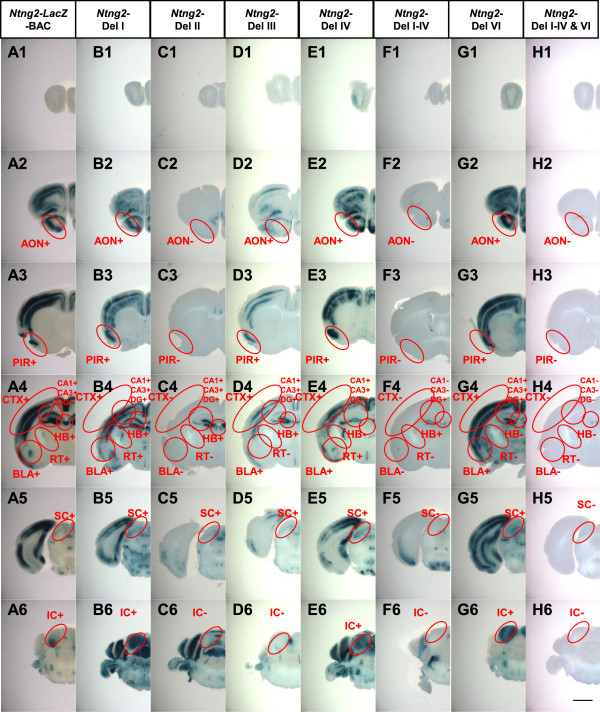
***LacZ *reporter expression profiles in the *Ntng2*-BAC deletion series.** Brains from transgenic mice at P21 carrying *Ntng2*-BAC-*LacZ* (column **A**), *Ntng2*-Del I **(B)**, II **(C)**, III **(D)**, IV **(E)**, I–IV **(F)**, VI **(G)**, and I–IV&VI **(H)** reporter constructs were sliced at 400 μm and stained for *LacZ* activity. Images of the stained slices are arranged from anterior (Top) to posterior (Bottom). Red circles indicate the regions evaluated for *LacZ* activity. Abbreviations of the brain regions are the same as those in Figure 1. The (+) and (−) symbols indicate that the given construct induced expression of *LacZ* at a frequency over the criterion (50% of transgenic founders) or not. Scale bar: 2.0 mm for all panels.

In the *Ntng2-LacZ*-BAC transgenic mice, *LacZ* expression was reproducibly detected in the cerebral cortex layers II/III, IV, and VI, anterior olfactory nucleus, piriform area layers III, hippocampal areas CA1 and CA3, dentate gyrus, basolateral amygdala nucleus, Bergmann glia cells, cerebellar nuclei, anterodorsal nucleus of the thalamus, reticular nucleus of the thalamus, habenula, superior colliculus, and inferior colliculus (Figure 4A; Additional file 3: Table S2). These expression patterns of *LacZ* in the *Ntng2-LacZ-*BAC transgenic mice were similar to those in the *Ntng2-LacZ*-KI mice (Figure 1L-V), indicating that the *Ntng2-LacZ-*BAC construct contained physiologically significant cis-regulatory elements for endogenous *Ntng2* expression.

To determine the critical regions for regulatory activity, the 195-kb region of the *Ntng2* locus was divided into six segments (I–VI) by considering the ECRs among species-mouse and human (Figure 3 and Additional file 4: Figure S2). This homology alignment was performed using a VISTA homology search program [33]. The adjacent gene, *Med27*, was also taken into consideration*. Ntng2* segments I-VI represent the −95 to −85 kb, −85 to −45 kb, −45 to −23 kb, −23 to −6 kb, and the end of *Ntng2* exon2 to +100 kb positions of the *Ntng2* TSS, respectively. The corresponding series of deletion constructs were referred to as *Ntng2-*Del I, II, III, IV, I–IV, VI, and I–IV&VI, respectively. Representative results from the *Ntng2* deletion series are shown in Figure 4. If the frequency of *LacZ* expression in a given brain area exceeded 50% of the sample, we considered that the construct carried a *cis-*element for a given brain area (Additional file 3: Table S2).

**Figure 5:**
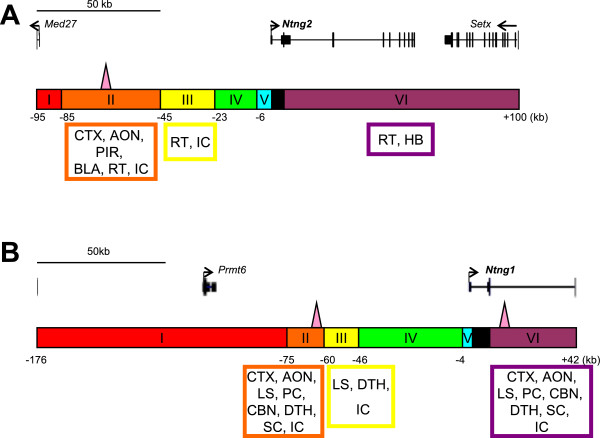
**Summary of regulatory regions required for *Ntng1 *and *Ntng2 *expression.** Genomic organization and genomic segments I–VI along with *Ntng2***(A)** and *Ntng1***(B)** gene organization are shown. Brain regions positively regulated by the genomic segments are indicated within the designated colored boxes at the bottom. The locations of *Ntng1* and *Ntng2* enhancers identified are indicated by pink triangles. The combinations of *cis*-elements that locate distantly and widely from the TSS determine differential expression patterns of the vertebrate-specific paralogs, *Ntng1* and *Ntng2*.

The deletion of segment I did not affect *LacZ* expression in any brain region examined (Figure 4B, Additional file 3: Table S2). Deletion of segment II led to a loss of *LacZ* expression in various brain areas, including the cerebral cortex, anterior olfactory nucleus, piriform area layers III, basolateral amygdala nucleus, reticular nucleus of the thalamus, and inferior colliculus (Figure 4C, Additional file 3: Table S2). Deletion of segment III led to a loss of *LacZ* expression in the reticular nucleus of the thalamus and inferior colliculus (Figure 4D, Additional file 3: Table S2). The deletion of segment IV did not affect *LacZ* expression in any brain area examined (Figure 4E, Additional file 3: Table S2). Large deletion of segments I-IV led to a loss of *LacZ* expression in CA1, CA3, and the superior colliculus, as well as brain areas affected by the deletion of a single segment II or III (Figure 4 F, Additional file 3: Table S2). The deletion of segment VI only affected *LacZ* expression in the reticular nucleus of the thalamus and habenula (Figure 4G, Additional file 3: Table S2). The combined deletion of segments I-IV and VI led to a complete loss of the transcriptional activity of the *Ntng2* promoter (Figure 4H, Additional file 3: Table S2). These data from the *Ntng2* BAC deletion series suggest that multiple enhancers are widely distributed upstream and downstream of the TSS site and that a major *cis-*element is located in segment II (from −85 to −45 kb of *Ntng2*) (Figure 5A). These findings also suggest that multiple *cis-*elements cooperate to regulate the transcriptional activities in several brain areas, such as hippocampus, anterodorsal thalamus, superior colliculus, cerebellar nuclei and Bergman glia (Additional file 3: Table S2).

### *Cis*-regulatory elements responsible for endogenous *Ntng1* expression

**Figure 6:**
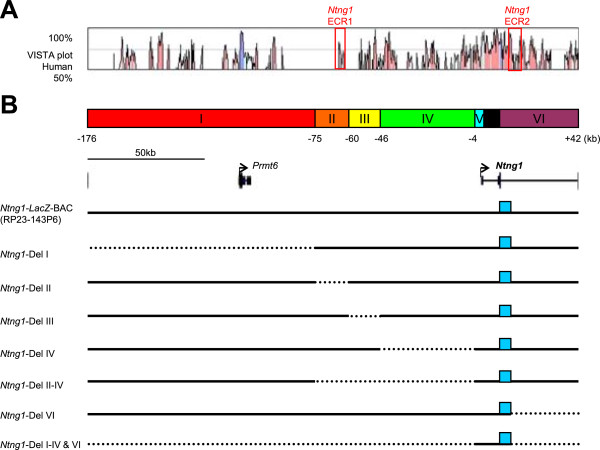
**Highly conserved sequences in *Ntng1 *loci and strategy for analyzing *cis*-regulatory elements in transgenic mice. (A)** Comparative genomic analysis of the mouse *Ntng1*-BAC clone (mouse Dec. 2011 [GRCm38/mm10] assembly; chr3:110,101,082 -110,318,433) by the VISTA genome browser. Percent nucleotide identities between mouse and human DNA sequences were plotted as a function of the position along the mouse sequence. Peaks of evolutionary-conserved exons of *Ntng1* and neighboring genes are shaded blue. Aligned regions with more than 70% identity over 100 bases are shaded pink. **(B)** Schematic diagrams of the *Ntng1* deletion series. The *Ntng1*-BAC sequences are divided into 6 segments (I–VI indicated in colored boxes) along with ECR distribution. Genomic organizations of the mouse *Ntng1* and neighbor gene loci are indicated. Short vertical lines indicate positions of exons and arrowheads indicate transcriptional directions. Broken and solid lines represent deleted and preserved regions in the BAC constructs, respectively. Blue boxes on the lines represent the reporter *LacZ* cassette inserted in each BAC construct. Note that the reporter *LacZ* cassette, ECRs, and minimal promoter sequences are not represented in accurate scales.

**Figure 7:**
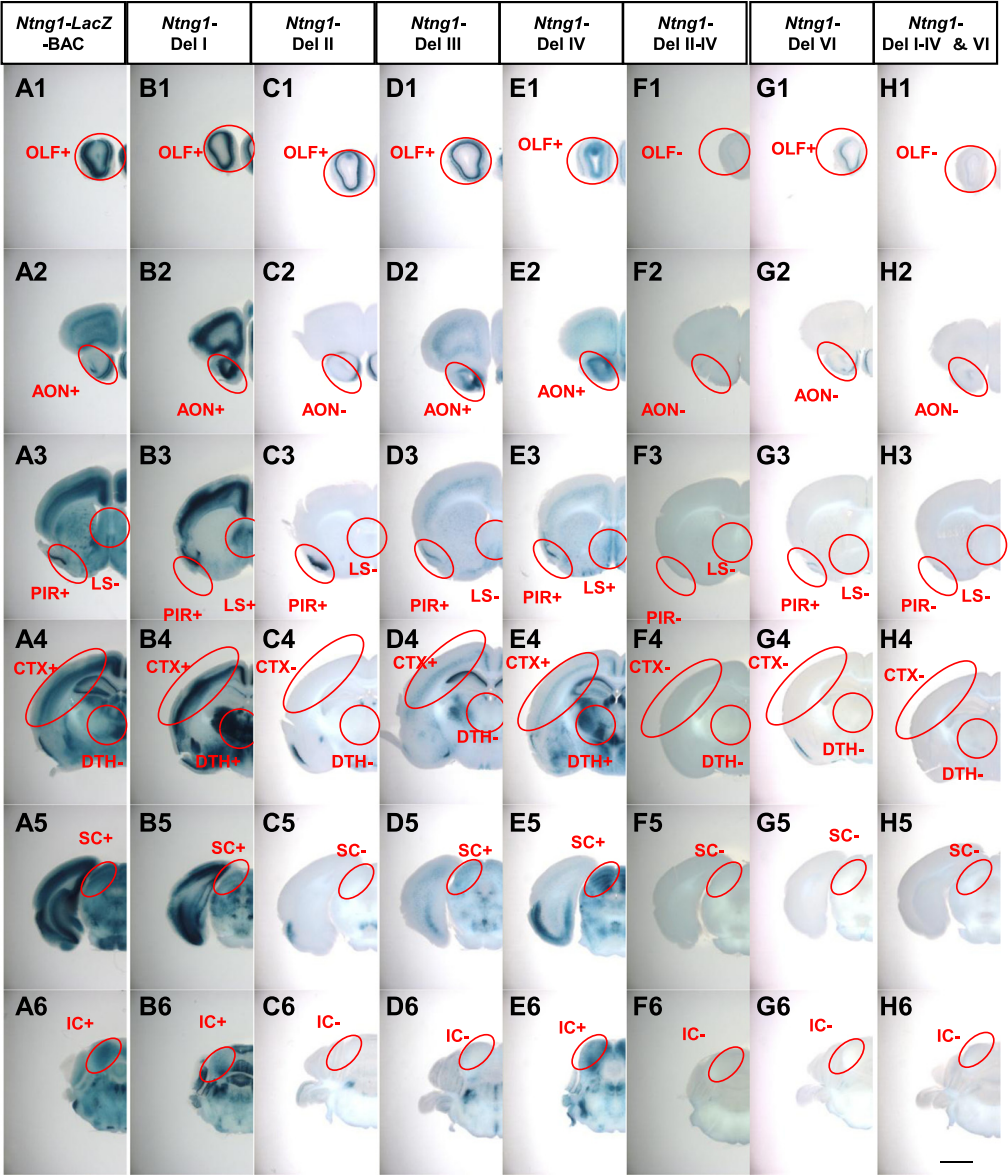
***LacZ *reporter expression profiles in the *Ntng1*-BAC deletion series.** Brains from transgenic mice at P21 carrying *Ntng1*-BAC-*LacZ* (column **A**), *Ntng1*-Del I **(B)**, II **(C)**, III **(D)**, IV **(E)**, II–IV **(F)**, VI **(G)**, and I–IV&VI **(H)** reporter constructs were sliced at 400 μm and stained for *LacZ* activity. Images of the stained slices are arranged from anterior (Top) to posterior (Bottom). Red circles indicate the regions evaluated for *LacZ* activity. Abbreviations of the brain regions are the same as those in Figure 1. The (+) and (−) symbols indicate that the given construct-induced expression of *LacZ* at the frequency over the criterion (50% of transgenic founders) or not. Scale bar: 2.0 mm for all panels.

Similar studies were performed for *Ntng1*. We selected a BAC clone (RP23-143P6) covering the 218-kb region of the locus between −174.9 kb and +42.5 kb of the *Ntng1* TSS (Figure 6), as a clone covering the longest region of putative transcriptional regulatory elements. The adjacent gene, *Prmt6,* locates 106-kb upstream of the *Ntng1* TSS. The *Ntng1*- BAC clone was inserted with an NLS-*LacZ*-pA cassette at the translation start site in exon 2. The procedures for generating and analyzing the transgenic mice were identical to those used for the *Ntng2* experiment. In transgenic mice carrying the *Ntng1-LacZ-*BAC construct, the *LacZ* expression patterns resembled those of the *Ntng1*-KI mice, although the expression levels differed significantly in the lateral septal nucleus, cerebellar nuclei, and dorsal thalamus (Figure 7A, Additional file 5: Table S3). Subsequent studies with a series of deletion constructs, however, suggested that the lack of expression in the thalamus did not indicate the lack of a *cis-*element in the thalamus.

To determine the critical regions for the regulation of *Ntng1* expression, we divided the 218-kb region of the *Ntng1* gene locus into six segments (I–VI) by considering the ECRs and adjacent gene, *Prmt6* (Figure 6A and Additional file 6: Figure S3). *Ntng1* segments I–VI represent the −176 to −75 kb, −75 to −60 kb, −60 to −46 kb, −46 to −4 kb, −4 kb to ATG of *Ntng1*, and at the end of *Ntng1* exon 2 to +42-kb positions of the *Ntng1* TSS, respectively. The corresponding deletion constructs were named *Ntng1-*Del I, II, III, IV, II–IV, VI, and I–IV&VI, respectively (Figure 6B). The results from the *Ntng1* deletion series are summarized in Additional file 5: Table S3 and are shown in Figure 7.

The deletion of segment I was not associated with a loss of *LacZ* expression in any brain area, but additional expression was observed in the lateral septal nucleus, cerebellar nuclei, and dorsal thalamus, resembling the expression patterns of endogenous *Ntng1*, and suggesting negative regulatory activity in this segment (Figure 7B, Additional file 5: Table S3). Deletion of segment II led to a marked loss of expression in most brain areas, including the deep cortical layer, anterior olfactory nucleus, lateral septum, Purkinje cells, cerebellar nuclei, dorsal thalamus, and superior and inferior colliculi (Figure 7C, Additional file 5: Table S3). Deletion of segment III led to a loss of expression in the lateral septal nucleus, dorsal thalamus, and inferior colliculus (Figure 7D, Additional file 5: Table S3). Deletion of segment IV did not show substantial effects (Figure 7E, Additional file 5: Table S3). Combined deletions of segments II-IV, however, led to an almost complete loss of transcriptional activity in all brain areas (Figure 7F, Additional file 5: Table S3). Interestingly, single deletion of segment VI also led to a loss of transcriptional activity in most brain areas, except for the olfactory bulb and piriform cortex (Figure 7G, Additional file 5: Table S3). Segment V alone did not show detectable activity (Figure 7H, Additional file 5: Table S3). These data from the *Ntng1* BAC deletion series suggest that multiple enhancers are widely distributed upstream and downstream of the TSS site and that significant *cis-*elements locate in segment II (from −75 to −60 kb of *Ntng1*) and segment VI (Figure 5B). These findings data also suggest that a negative regulator(s) for the lateral septal nucleus, cerebellar nuclei, and dorsal thalamus is located in segment I (Additional file 5: Table S3).

Unlike the *Ntng2-*BAC constructs, the *Ntng1-*BAC constructs showed greater variability in their reporter gene expression patterns. The variability may partly depend on the intergenic recombination of BAC constructs. For example, in the *Ntng1* BAC deletion series, 5’ and 3’ BAC end sequences were maintained in transgenic mice at a frequency of 31/60 (52%). The ratio was significantly lower than that in the *Ntng2* BAC deletion series (42/59, 71%).

### Enhancer activities of *Ntng1* and *Ntng2* ECRs

**Figure 8:**
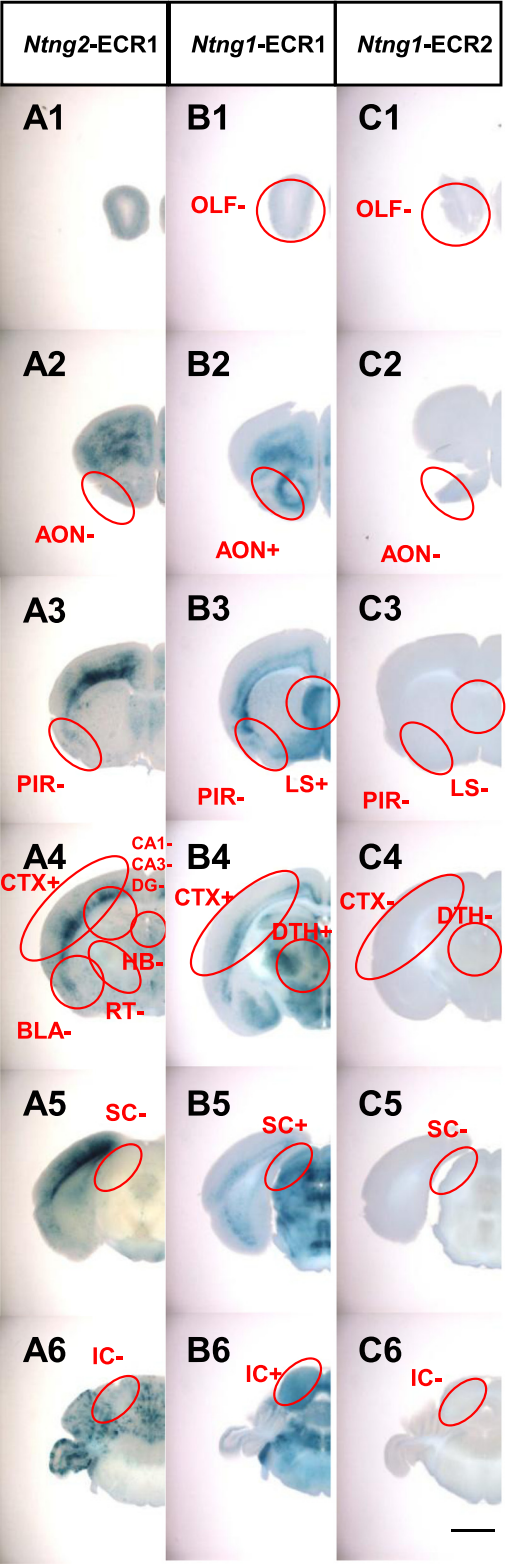
***LacZ *reporter expression profiles in the *Ntng1 *and *Ntng2 *enhancer assays.** Brains from transgenic mice at P21carrying *Ntng2*-ECR1 (**A** column), *Ntng1*-ECR1 **(B)**, and *Ntng1*-ECR2 **(C)** reporter constructs were sliced at 400 μm and stained for *LacZ* activity. Images of the stained slices are arranged from anterior (Top) to posterior (Bottom). Red circles guide the regions evaluated for *LacZ* activity. Abbreviations of the brain regions are the same as those in Figure 1. The (+) and (−) symbols indicate that the given construct-induced expression of *LacZ* at the frequency over the criterion (50% of transgenic founders) or not. Scale bar: 2.0 mm for all panels.

Findings from the *Ntng1* and *Ntng2* BAC deletion series suggest that segment II in the *Ntng2* and segment II and VI in the *Ntng1* carry physiologically significant *cis-*regulatory elements. Based on comparisons among mouse, rat, human, chimp, rhesus, cow, dog, and chicken genomes, we focused on the most prominent ECRs; i.e., *Ntng2-*ECR1, *Ntng1-*ECR1, and *Ntng1*-ECR2 (Additional files 4 and 6 Figures S2-S3). *Ntng2-*ECR1 in segment II locates at the −68-kb of TSS. *Ntng1*-ECR1 and ECR2 locate at the −65-kb of TSS in segment II and the +11-kb of TSS in segment VI, respectively. We evaluated the enhancer activities of these ECRs using a transgenic mouse enhancer assay in which the activity of each sequence was assessed through a *LacZ* reporter gene coupled to a minimal *Hsp68* promoter. Three constructs, *Ntng2*-ECR1*-LacZ, Ntng1-*ECR1*-LacZ*, and *Ntng1-*ECR2*-LacZ* were generated and injected into the pronuclei of fertilized eggs. X-gal staining was performed to analyze the expression of *LacZ* in transgenic founders at P21. The results of these ECR-*LacZ* transgenic mice are shown in Figure 8 and Additional file 7: Table S4.

Transgenic mouse lines carrying the *Ntng2-*ECR1-*LacZ* construct had *LacZ* expression in the deeper cerebral layer and cerebellum (Figure 8A, Additional file 7: Table S4). The findings suggest that *Ntng2-*ECR1, located at the −68 kb region in *Ntng2* segment II, contains an enhancer responsible for the transcriptional regulation of *Ntng2* in the cerebral cortex.

Transgenic lines carrying the *Ntng1-*ECR1-*LacZ* construct had *LacZ* expression in the regions of endogenous *Ntng1* expression, such as the cerebral cortex layer V, anterior olfactory nucleus, lateral septal nucleus, Purkinje cells, dorsal thalamus, superior colliculus, and inferior colliculus (Figure 8B, Additional file 7: Table S4). In transgenic lines carrying the *Ntng1*-ECR2*-LacZ* construct, however, no significant *LacZ* expression was detected in any brain region (Figure 8C, Additional file 7: Table S4). Therefore, these findings suggest that *Ntng1*-ECR1, located at the −65-kb region in *Ntng1* segment II, but not *Ntng1-*ECR2, contains a physiologically significant enhancer responsible for the transcriptional regulation of endogenous *Ntng1*.

## Discussion

In the present study, we investigated the mechanisms responsible for the complementary expression patterns of *Ntng1* and *Ntng2* in the central nervous system. Analyses of reporter gene expression patterns for both genes using *Ntng1*- and *Ntng2*-*LacZ-*KI mice confirmed the largely non-overlapping expression patterns of *Ntng1* and *Ntng2*. Analysis of the expression patterns in KI alleles in the presence or absence of netrin-G1 and netrin-G2 functional proteins allowed us to exclude the possibility that a feedback (or feedforward) mechanism mediates the characteristic expression patterns of these genes. We next examined the *cis*-acting transcriptional regulatory mechanisms of *Ntng1* and *Ntng2* using BAC transgenic mice and transgenic enhancer assays. The results obtained from a series of BAC clones carrying systematic deletions revealed a complex regulatory architecture that depended on a diverse array of segments scattered over a 100-kb range of the locus in both genes. The *Ntng2*-segment II region contained major elements responsible for most brain regions. The *Ntng1*-segment II and VI regions cooperatively regulated *Ntng1* expression in most brain regions. Furthermore, we successfully identified a 1.8-kb enhancer of *Ntng2* and a 2.0-kb enhancer of *Ntng1* located at ECR regions ~60 kb upstream from TSSs of both genes. These findings suggest that the complementary expression patterns of *Ntng1* and *Ntng2* are determined by transcriptional *cis-*elements widely scattered around the TSSs of these genes.

The high-resolution and highly sensitive analysis using *LacZ*-KI mice revealed a detailed picture of the complementary expression patterns of *Ntng1* and *Ntng2* in young adult mouse brains. *Ntng1* is most abundantly expressed in brain regions involved in processing the early phases of sensory signal integration, such as the dorsal thalamus, superior colliculus, inferior colliculus, and olfactory bulb. The superior colliculus and lateral geniculate nuclei of the thalamus receive inputs from the retina and relay processed visual signals to the cerebral cortex. The inferior colliculus and medial geniculate nuclei of the thalamus process auditory signals, and the olfactory bulbs process olfactory signals. The dorsal thalamus is involved in processing all sensory signals, including secondary olfactory signals. Therefore, the regions that express *Ntng1* are mainly involved in processing bottom-up signals. In contrast, *Ntng2* is prominently expressed in the cortical layers, hippocampal circuits, habenula, claustrum, endopiriform nuclei, and reticular nuclei of the thalamus. These sites are involved in later stages of information processing, including top-down signals. The hippocampus plays a crucial role in several forms of learning and memory [34-38]. The frontal cortex is also involved in working memory [39-41]. Crick and Koch [42] proposed a role for the claustrum in integrated conscious perception as well as a role for the reticular nuclei of the thalamus in controlling attention by regulating sensory signal processing (i.e., the Searchlight hypothesis; [43]). The habenular complex has important roles in modulating learning, memory, and attention [44,45], and habenular dysfunction might be involved in schizophrenia and bipolar disorders [46]. Thus, the mechanisms that underlie the complementary expression patterns of *Ntng1* and *Ntng2* have had a large impact on the evolution of higher brain functions in vertebrates, particularly mammals.

Using the *Ntng2*-ECR1-*LacZ* construct, we induced reporter expression in the deep cortical layer. The BAC clone with a deletion of sequences containing the ECR1 failed to induce the expression of *LacZ* in the cortex. These findings suggest that the ECR1 at the 68-kb upstream region from the *Ntng2* TSS works as a deep cortex layer-specific enhancer. The *Ntng2*-ECR1 sequences constituted a DNase I-hypersensitive site in adult mouse cortex (UCSC Genome Browser). DNase I-hypersensitive sites are regions of accessible chromatin, which are sensitive to cleavage by the DNase I enzyme and are markers of *cis-*regulatory elements [47]. Furthermore, we investigated transcription factors potentially involved in the 1.8-kb *Ntng2*-ECR1 sequences, using DiAlign TF [48] (Genomatix [ http://www.genomatix.de/index.html]). DiAlign TF is a web-based tool for predicting transcription factor binding sites using cross-species comparisons. The *Ntng2*-ECR1 sequences were highly conserved among mouse, rat, human, chimpanzee, rhesus macaque, cow, dog, and chicken (Additional file 4: Figure S2), and contained 17 transcription factor binding sites within the sequences (Additional file 8: Figure S4). Among the sites, it is noteworthy the presence of CREB (cAMP response element–binding protein) site within the *Ntng2*-ECR1. CREB have crucial roles in activity-dependent transcriptional regulation in neurons [49]. We observed the activity-dependent increase in *Ntng2* gene expression by age and behavioral experience in mice (unpublished observation by Pavel Prosselkov and SI). The CREB site in *Ntng2*-ECR1 may have a crucial role in the activity-dependent *Ntng2* expression and circuit plasticity. Taken together, these findings suggest that the *Ntng2*-ECR1 sequences act as an enhancer in adult mouse cortex.

In the case of *Ntng1,* we observed cooperative roles of *Ntng1* segment II and VI for transcriptional regulation in most brain regions, suggesting that the *Ntng1* regulatory mechanisms are highly complex. This observation may in part explain the reason of the longer genomic structure of *Ntng1* (362 Kb, between the 1st and last exons) relative to that of *Ntng2* (53 kb). Despite the complexity, however, we successfully identified enhancer activity in the *Ntng1*-ECR1 sequences, located at the −65-kb region in *Ntng1* segment II. The *Ntng1*-ECR1 sequences are relatively well conserved among mammals, but not in chicken (Additional file 6: Figure S3). DiAlign TF analysis based on comparisons between mouse and dog predicted sites for a limited number of transcription factors in the *Ntng1*-ECR1 (Additional file 9: Figure S5). These findings suggest that the *cis-*mechanism for *Ntng1* has a radical evolutional history relative to that of *Ntng2*-ECR1.

In the *Ntng1* BAC deletion study, *Ntng1*-Del II and *Ntng1*-Del VI transgenic mice showed a highly restricted expression pattern in the olfactory bulb (mitral cells) and piriform cortex. Thus, the expression of *Ntng1* is separately regulated in the olfaction and other sensory modality pathways. The *cis-*mechanisms of *Ntng1* make a physiologically significant contribution to elaborating the perception of multiple modalities in vertebrates/mammals. Practically, the *Ntng1*-Del II can be used as a useful transgenic vector in mice. Indeed, using the *Ntng1*-Del II, we successfully established an olfactory bulb and piriform cortex-specific *Cre* mouse line (Additional file 10: Figure S6).

Recent genome studies reported the whole genome sequences of the ascidian and amphioxus of chordates and the lamprey of early vertebrates [50-52]. The ancestral single *Ntng* ortholog (EntrezGene ID: 100178743) emerged in the ascidian genome in the evolutionary process. *Ntng1* (Ensembl Gene ID: ENSPMAG00000002937) and *Ntng2* (Ensembl Gene ID: ENSPMAG00000003799) are found in the lamprey genome, which underwent the first round of whole genome duplication after divergence from chordates. Therefore, it is likely that ancestral *Ntng* was divided into genes, *Ntng1* and *Ntng2*, during the first round of whole genome duplication. It is noteworthy that *Ntng1*-ECR1 and *Ntng2-*ECR1 are equally distant from the TSSs. We argue that the *cis-*elements of *Ntng1* and *Ntng2* co-evolved in accordance with the duplication-degeneration-complementation model [53,54]. Differential behavioral phenotypes of *Ntng1-*KO and *Ntng2-*KO mice [13] clearly reveal the remarkable molecular evolution of *Ntng1* and *Ntng2*.

## Conclusions

Our findings suggest that the complementary expression patterns of *Ntng1* and *Ntng2* are determined by combinations of *cis*-regulatory elements that are widely scattered in these loci. The *cis*-regulatory elements in adult mouse brain characterized in this study will help facilitate the development of novel genetic tools for functionally dissecting neural circuits to better understand vertebrate brain function.

## Methods

### Gene-targeted mice

*Ntng1*-*LacZ*-KI and *Ntng2-*KO mice were described previously [12]. Using the same target sequences, we generated *Ntng2*-*LacZ*-KI mice and *Ntng1-tauGFP-*KI mice, which carried either a NLS-*LacZ*-polyA cassette or a tau and green fluorescent protein (GFP) fusion gene-polyA cassette within the exon 2 coding regions of *Ntng1* or *Ntng2*. Mice with these mutations do not express endogenous netrin-G1 or netrin-G2. To genotype the KO and KI mice, DNA was extracted from the tails using a REDExtract-N-Amp Tissue PCR Kit (Sigma-Aldrich Chemical Co., St. Louis, MO, USA). The *LacZ* transgene was detected by polymerase chain reaction (PCR) of the tail DNA with 30 cycles of 95°C for 1 min, 60°C for 1 min, and 72°C for 1 min. A pair of primers (Primer Nos. 1 and 2; Additional file 11: Table S5) was used to amplify the 515-bp *LacZ* region. The *tau-GFP* transgene was detected by PCR of the tail DNA under the same conditions. A pair of primers (Primer Nos. 3 and 4; Additional file 11: Table S5) was used to amplify the 303-bp *tau-GFP* region. The mice were maintained in the RIKEN animal facility. Mouse genotypes were determined by PCR using primers (Primer Nos. 1, 2, 5–9; Additional file 11: Table S5). Primer pairs of *Ntng1* check up2/*Ntng1* check down2 and *LacZ1/LacZ2* yielded 199-bp and 515-bp fragments from the wild-type and *Ntng1* KO allele, respectively. *Ntng2* check up, *Ntng2* check up2, and *Ntng2* check down3 were used for genotyping the *Ntng2* KO line. *Ntng2* check up/*Ntng2* check down3 and *Ntng2* check up2/*Ntng2* check down3 amplified 936-bp and 407-bp fragments from the wild-type and *Ntng2* KO allele, respectively. All experimental protocols were approved by the RIKEN Institutional Animal Care and Use Committee.

### BAC clones

Mouse BAC clones containing *Ntng1* (RP23-143P6) and *Ntng2* (RP23-417D10) from the C57BL/6 J mouse BAC library were purchased from the BACPAC Resource Center [ http://bacpac.chori.org/home.htm]. In this study, BAC clones RP23-143P6, and RP23-417D10 were renamed *Ntng1*-BAC, and *Ntng2*-BAC, respectively. *Ntng1*-BAC covered a 218-kb region (mouse Dec. 2011 [GRCm38/mm10] assembly; chr3:110,101,082 -110,318,433) of the locus, representing the 176-kb upstream (−176 kb) and 42-kb downstream (+42 kb) sequences of *Ntng1* TSS. *Ntng2*-BAC covered a 195-kb region (mouse Dec. 2011 [GRCm38/mm10] assembly; chr2:29,153,280-29,347,847) of the locus, representing the sequences of *Ntng2* TSS from −95 kb to +100 kb.

### Modification of BAC clones

BAC clones were modified using the Red/ET recombination technique (Gene Bridges, Dresden, Germany), which is based on homologous recombination aided by the inducible Red/ET recombination machinery. The NLS-*LacZ*-pA-FRT5-*Kan*-FRT5 reporter cassette contains NLS-*LacZ*-pA and a prokaryotic promoter-driven kanamycin resistance gene (*Kan*) flanked by FRT5 sites [55,56]. To construct this cassette, the NLS-*LacZ*-pA cassette from the BIN*LacZ* plasmid [57] was subcloned into an FRT5-flanked *Kan* vector (pCR-FRT5-*Kan*-FRT5) that was generated by inserting the PCR-amplified FRT5-flanked *Kan* cassette into a pCR-Blunt II-TOPO plasmid (Invitrogen, Carlsbad, CA, USA). The NLS-*LacZ*-pA-FRT5-*Kan*-FRT5 reporter cassette was amplified by PCR using Phusion High-Fidelity DNA Polymerase (Finnzymes, Espoo, Finland). The primer sequences used (Primer Nos. 10–13) are shown in Additional file 11: Table S5. For in-frame replacement of the exon containing the *Ntng1* or *Ntng2* translation initiation codon (ATG) by the reporter cassette in the two BAC clones, each primer was designed to have 5’ 60 nucleotide sequences corresponding to the BAC sequences and 3’ 20 nucleotide sequences corresponding to the reporter cassette sequences. The PCR products were treated with *Dpn*I to selectively digest the template plasmids and then purified by ethanol precipitation. *E. coli* cells carrying BAC were transformed with a Red/ET expression plasmid, pSC101-BAD-gbaA (Gene Bridges), and recombination with the reporter cassette (NLS-*LacZ*-pA-FRT5-*Kan*-FRT5) was subsequently induced. Successfully recombined colonies were identified by screening for kanamycin resistance, followed by PCR to ensure homologous recombination. The *Kan* cassette was subsequently removed from the recombined BAC clones by introducing the Flp recombinase expression plasmid, 706-FLP (Gene Bridges), into the bacterial cells. The resulting BAC transgene construct was verified by PCR, restriction fragment length polymorphisms, and DNA sequencing analyses.

A BAC deletion series was also generated using the Red/ET recombination system. The pCR-FRT-*Amp*-FRT plasmid was generated by inserting the PCR-amplified FRT-flanked *Amp* cassette into a pCR-Blunt II-TOPO plasmid (Invitrogen). FRT-flanked *Amp*-targeting fragments were amplified by PCR using Phusion High-Fidelity DNA Polymerase, 80-mer primers (Primer Nos. 17–33; Additional file 11: Table S5), and the pCR-FRT-*Amp*-FRT plasmid as a template. Subcloning from BAC constructs for *Ntng1*-Del I-IV &VI and *Ntng2*-Del I-IV &VI was also performed using the Red/ET recombination system. Fragments from −4 kb (*Ntng1*) or −6 kb (*Ntng2*) of TSSs to the 3' end of the NLS-*LacZ*-pA cassette from *Ntng1*-*LacZ*-BAC and *Ntng2*-*LacZ*-BAC constructs were subcloned into a pDEST R4-R3 vector (Invitrogen), containing the ampicillin resistance gene (*Amp*) and pUC origin of replication. The pDEST R4-R3 linear vector fragment was amplified by PCR using Phusion High-Fidelity DNA Polymerase (Finnzymes) and primers (Primer Nos. 14–16; Additional file 11: Table S5).

The *Ntng1*-Del II-*Cre* BAC transgenic construct was also generated using the Red/ET system. The NLS*-Cre*-pA cassette was replaced the *LacZ* cassette of *Ntng1*-Del II construct.

Modified BAC clones were propagated in 2 × 400 ml of liquid culture and purified using a QIAGEN Large-Construct Kit (QIAGEN, Hilden, Germany) according to the manufacturer’s protocol. Correctly modified BACs were verified by conventional and pulsed-field gel analysis of restriction digests to confirm expected banding patterns and direct BAC sequencing at a vector-insert junction and *LacZ* cassette. All BAC reporter constructs were linearized by *Asc*I digestion and purified for microinjection on sepharose columns as described previously [58].

### Generation of *Ntng*-ECR-Hsp-*LacZ* constructs

*Ntng*-ECR-*Hsp68-LacZ* transgenes were generated as follows. PCR products corresponding to genomic sequences of *Ntng1*-ECR1 (2.0 kb), *Ntng1*-ECR2 (5.5 kb), and *Ntng2*-ECR1 (1.8 kb) were amplified using *Ntng1*- or *Ntng2*-BAC DNA as the template, Phusion High-Fidelity DNA Polymerase, and primers (Primer Nos. 34–39; Additional file 11: Table S5). Each PCR product was ligated into the ASS-*Hsp-LacZ-*pA vector in the forward orientation to create ECR-*Hsp-LacZ* transgenes [59,60]. All ECR plasmids were verified by analysis of restriction enzyme digestion and DNA sequencing. Plasmid DNAs were purified using NucleoBond PC 500 (Macherey-Nagel) and digested with *Not*1. For injection, an ECR-*Hsp-LacZ* cassette without a vector fragment was isolated by agarose gel electrophoresis and purified using QIAEX II (QIAGEN). All ECR-Hsp-*LacZ* constructs were verified by analysis of restriction digests and direct sequencing.

### Generation of transgenic mice

The purified DNA fragment (1.0 ng/μl) was injected into the pronuclei of fertilized eggs of C57BL/6 J × DBA/2 J F1 hybrid mice to generate transgenic mice. To genotype the transgenic mice, DNA was extracted from mouse tail samples collected at P10 using the REDExtract-N-Amp Tissue PCR Kit (Sigma Chemical Co.), and *LacZ* sequences were detected by PCR as described above. *Cre* transgenic mice were genotyped by PCR, using the primers (Primer Nos. 42 and 43; Additional file 11: Table S5), producing a 108-bp fragment from the *Cre* allele. For BAC transgenic organisms, the presence of pBACe3.6 BAC vector sequences immediately upstream or downstream of the Asc I site was further examined by PCR to minimize the possibility that large deletions occurred in the BAC integrated into the chromosome. *LacZ* expression analyses were performed for independent transgenic founders.

### X-gal staining

For X-gal staining of the brain, mice were anesthetized by an intraperitoneal injection of 2.5% 2,2,2-tribromoethanol at P21 and their brains were fixed with 40 ml of 4% paraformaldehyde in phosphate-buffered saline (PBS) via transcardial perfusion at 4°C for 10 min after washing out the blood with physiologic saline. The fixed brains were sliced (thickness, 400 μm) using a Microslicer DTK-1000 (Dosaka EM, Kyoto, Japan) and placed in X-gal staining solution (0.1 M phosphate buffer [pH 7.5], 20 mM Tris–HCl [pH 7.5], 5 mM K_3_Fe(CN)^6^, 5 mM K_4_Fe(CN)^6^, 2 mM MgCl_2_, 1 mg/ml X-gal) for over 15 h at 37°C.

For X-gal staining of thin sections, fixed brains were removed and postfixed in the same fixative for 2 h. The brains were cryoprotected overnight in 15% sucrose in PBS and then overnight in 30% sucrose in PBS at 4°C. They were then mounted in Tissue-Tek OCT compound (Sakura Finetek, Torrance, CA, USA), and stored at −80°C until sectioned. Cryostat sections (25 μm) were cut and thaw-mounted on MAS-coated slides (Matsunami Glass, Osaka, Japan). The slices were dried at room temperature and then stained overnight at 37°C in X-gal staining solution. After a brief rinse in PBS, the tissue was counterstained with hematoxylin (Sakura Finetek, Japan) for 2 min. After a brief rinse in PBS, the tissue was dehydrated in an ethanol series (70%, 85%, 95%, and 100% ethanol), dehydrated in xylene, and mounted with Eukitt (O. Kindler GmbH &*Co.,* Freiburg, Germany). Images of the entire brain sections were captured using a NanoZoomer RS slide scanner (Hamamatsu Photonics, Shizuoka, Japan) at 200-fold magnification.

### Comparative genome analysis

Comparative genome analysis of the mouse *Ntng1*- and *Ntng2*-BAC sequences was performed using the VISTA browser [ http://genome.lbl.gov/vista/index.shtml] [32]. Analysis of *Ntng1* and *Ntng2* loci covered the *Ntng1*-BAC (RP23-143P6) sequence (mouse Dec. 2011 [GRCm38/mm10] assembly; chr3:110,101,082 -110,318,433) and *Ntng2*-BAC (RP23-417D10) sequence (mouse Dec. 2011 [GRCm38/mm10] assembly; chr2:29,153,280-29,347,847), respectively, as the reference sequences. The reference sequences included *Ntng1* (NM_133488.1), *Prmt6* (NR_024139.1), *Ntng2* (NM_133501.1), *Setx* (NM_198033.2), and *Med27* (NM_026896.4). Multiple alignments of *Ntng1* and *Ntng2* regions in mice versus those of *Ntng1* and *Ntng2* regions in other vertebrates were performed using the shuffle-LAGAN program [61]. Multiple alignments of the following assemblies were used for this analysis: mouse (Dec. 2011 [GRCm38/mm10] assembly), rat (Mar. 2012 [RGSC 5.0/rn5] assembly), human (Feb. 2009 [GRCh37/hg19] assembly), chimpanzee (Feb. 2011 [CSAC 2.1.4/panTro4] assembly), rhesus macaque (Oct. 2010 [BGI CR_1.0/rheMac3] assembly), cow (Oct. 2011 [Baylor Btau_4.6.1/bosTau7] assembly), dog (Sep. 2011 [Broad CanFam3.1/canFam3] assembly), chicken (Nov. 2011 [ICGSC Gallis_gallus-4.0/galGal4] assembly), and zebrafish (Jul. 2010 [Zv9/danRer7] assembly). We used the default VISTA parameter settings. The conservation rate between mice and the other vertebrates was plotted as percentages, and only the genomic regions with 50%–100% conservation are shown in the Additional file 4: Figures S2 and Additional file 6: Figure S3.

Potential transcription factor-binding sites within *Ntng2*-ECR1 and *Ntng1*-ECR1 were predicted with the DiAlign TF [48] (Genomatix [ http://www.genomatix.de/index.html]). Selected groups for solution parameters of DiAlign TF were as follows: MatInspector library Version 9.1; matrix group, general core promoter elements and vertebrates; matrix similarity, optimized for function.

## Abbreviations

BAC: Bacterial artificial chromosome; kb: Kilo base; Ntng1: Netrin-G1; Ntng2: Netrin-G2; KO: Knock-out; ECR: Evolutionarily conserved region; LacZ: *E.coli beta*-galactosidase; KI: Knock-in; TSS: Transcription start site; NLS: Nuclear localization signal; pA: Polyadenylation signal; X-gal: 5-bromo-4-chloro-3-indolyl-β-d-galactoside; P21: Postnatal day 21; OR: Olfactory receptor; GFP: Green fluorescent protein; PCR: Polymerase chain reaction; Kan: Kanamycin resistance gene; ATG: Translation initiation codon; Amp: Ampicillin resistance gene; PBS: Phosphate-buffered saline; BR: Brain regions; AD: Anterodorsal nucleus of the thalamus; AON: Anterior olfactory nucleus; BC: Bergmann glia cells; BLA: Basolateral amygdala nucleus; CA1: Cornu ammonis 1 field of the hippocampus; CA3: Cornu ammonis 3 field of the hippocampus; CBN: Cerebellar nuclei; CLA: Claustrum; CN: Cochlear nuclei; CTX: Cerebral cortex; CU: Cuneate nucleus; DG: Dentate gyrus; DMX: Dorsal motor nucleus of the vagus nerve; DTH: Dorsal thalamus; ENT: Entorhinal area; Epd: Endopiriform nucleus, dorsal part; HB: Habenula; IC: Inferior colliculus; isl: Olfactory tubercle, islands of Calleja; IO: Inferior olivary complex; LA: Lateral amygdala nucleus; LRN: Lateral reticular nucleus; LS: Lateral septal nucleus; OLFgl: Olfactory bulb, glomerular layer; OLFmi: Olfactory bulb, mitral layer; PAG: Periaqueductal gray, dorsal division; PC: purkinje cell; PIR: Piriform area; PG: Pontine gray; PRT: Pretectal region; PSV: Principal sensory nucleus of the trigeminal; RAmb: Midbrain raphe nuclei; RN: Red nucleus; RT: Reticular nucleus of the thalamus; SC: Superior colliculus; SPV: Spinal nucleus of the trigeminal; STN: Subthalamic nucleus; SUB: Subiculum; SUM: Supramammillary nucleus; TRN: Tegmental reticular nucleus; TT: Taenia tecta; III: Oculomotor nucleus; V: Trigeminal motor nucleus; VII: Facial motor nucleus; XII: Hypoglossal nucleus.

## Competing interests

The authors declare that they have no competing interests.

## Authors’ contributions

KY performed the experiments. KY and SI designed the experiments and analyzed data. SN provided the *Ntng1* and *Ntng2* gene-targeted mice. KY and SK generated and analysed a *Cre* transgenic mouse line. TO analyzed data. KY and SI wrote the manuscript. All authors have read and approved the manuscript.

## Supplementary Material

Additional file 1: Table S5 Primer sequences. Click here for file

Additional file 2: Figure S1 *LacZ*-stained brain sections of *Ntng1* and *Ntng2 LacZ* KI mice at high magnification. (A-E) X-gal staining of *Ntng1*-*LacZ* KI mice. (F-J) X-gal staining of *Ntng2*-*LacZ* KI mice. (A-C, E-H, J) Coronal sections (25 μm) at P21. (D, I) Horizontal sections (25 μm) at P21. Sections were lightly counterstained with hematoxylin. In the anterior olfactory nucleus, *Ntng1* was expressed in the neurons of dorsal-, lateral-, medial-part, and *Ntng2* was expressed in the neurons of the external part. In the piriform cortex, *Ntng1* was expressed in the semilunar neurons of layer II, whereas *Ntng2* was expressed in the pyramidal and polymorphic neurons of layer III. In the cerebral cortex, *Ntng1* was expressed in layer V neurons, whereas *Ntng2* was expressed in neurons in layers II/III, IV, and VI. In the entorhinal area, *Ntng1* was expressed in layer II of the lateral entorhinal area (LEC) and layer III throughout the entorhinal area, and *Ntng2* was expressed in layer II of the medial entorhinal area (MEC) and layer III of the LEC. In the amygdala, *Ntng1* was expressed in the lateral amygdala nucleus (LA), whereas *Ntng2* was expressed in the basolateral amygdala nucleus (BLA). Scale bar: 1.0 mm for all panels. Click here for file

Additional file 3: Table S2 Summary of *LacZ* expression domains observed in the *Ntng2* deletion series. The ratio of transgenic mice with reproducible reporter expression per all independent founders in regions expressing endogenous *Ntng2* is shown. Blue cells: reproducible expression in more than half of all independent transgenic founders in the given brain region. Click here for file

Additional file 4: Figure S2 Evolutionarily conserved region (ECR) in the mouse *Ntng2* locus. Analysis of the *Ntng2* locus covered by the *Ntng2*-BAC (mouse Dec. 2011 [GRCm38/mm10] assembly; chr2: 29,153,280-29,347,847). Comparative genomic analysis of the mouse *Ntng2*-BAC sequence by the VISTA genome browser ( http://genome.lbl.gov/vista/index.shtml). Percent nucleotide identities between mouse and other species (rat, human, chimp, rhesus, cow, dog, chicken, and zebrafish; from top to bottom) are plotted as a function of the position along the mouse sequence. Peaks of evolutionarily conserved overlapping exons of *Ntng2* and neighboring genes are shaded blue. Aligned regions with more than 70% identity over 100 bases are shaded pink. *Ntng2* ECR1, indicated by the red rectangle, represents the most highly conserved region and locates within segment II (Figures 3 and 5). Click here for file

Additional file 5: Table S3 Summary of *LacZ* expression domains observed in the *Ntng1* deletion series. The ratio of transgenic mice with reproducible reporter expression per all independent founders in regions of endogenous *Ntng1* expression is shown. Blue cells: reproducible expression in more than half of all independent transgenic founders in the given brain region. Light blue cells: although the ratio of reproducible expression did not fulfill the criteria, the highly restricted expression pattern might be significant. Click here for file

Additional file 6: Figure S4 Multiple sequence alignment and candidate transcription-factor binding sites in *Ntng2*-ECR1. Sequence comparisons of the *Ntng2* enhancer *Ntng2*-ECR1 sites between mouse, rat, human, chimp, rhesus, cow, dog, and chicken species. Putative transcription factor binding sites are highlighted in the differential colors, respectively. Seventeen potential highly conserved binding sites were identified (searched results are from DiAlign TF). Click here for file

Additional file 7: Table S4 Summary of *LacZ* expression domains observed in the *Ntng2* and *Ntng1* enhancer analysis series. The ratio of transgenic mice with reproducible reporter expression in regions expressing endogenous *Ntng2* (left) and *Ntng1*(right) is shown. Blue cells: the results were consistent with those from the deletion series. Click here for file

Additional file 8: Figure S3 ECR in the mouse *Ntng1* locus. Analysis of the *Ntng1* locus covered by the *Ntng1*-BAC (mouse Dec. 2011 [GRCm38/mm10] assembly; chr3:110,101,082 -110,318,433) by the VISTA genome browser ( http://genome.lbl.gov/vista/index.shtml). Percent nucleotide identities between mouse and other species (rat, human, chimp, rhesus, cow, dog, chicken, and zebrafish; from top to bottom) are plotted as a function of the position along the mouse sequence. Peaks of evolutionary conservation overlapping exons of *Ntng1* and neighboring genes are shaded blue. Aligned regions with more than 70% identity over 100 bases are shaded pink. *Ntng1* ECR1 and ECR2, indicated by the red rectangles, represent one of the most highly conserved regions and locate within segments II and VI, respectively (Figures 5 and 6). Click here for file

Additional file 9: Figure S5 Multiple sequence alignment and candidate transcription-factor binding sites in *Ntng1*-ECR1. Sequence comparisons of the *Ntng1* enhancer *Ntng1*-ECR1 sites between mouse, rat, human, chimp, rhesus, cow, and dog species. Putative transcription factor binding sites are highlighted in the differential colors, respectively . Eight potential highly conserved binding sites were identified (searched results are from DiAlign TF). Click here for file

Additional file 10: Figure S6 Recombination patterns in *Ntng1*-Del II-*Cre* : Rosa-NLSLacZ mice. (A-H) *LacZ* activies (blue signals) of *Ntng1*-*Del II-Cre* :Rosa-NLSLacZ [44] mice. Coronal sections (A-F: 400 μm, G-H: 25 μm) were stained with X-gal solution. Sections were lightly counterstained with hematoxylin. Red circles indicated the region-specific *LacZ* activities. In the olfactory areas, *Cre* was highly expressed in the olfactory bulb mitral cells. In the piriform cortex, *Cre* was expressed in the semilunar neurons of layer II. Scale bar: 2.0 mm for A-F panels, 500 μm for G-H panels. Click here for file

Additional file 11: Table S1. Summary of *LacZ* expression in *Ntng1* and *Ntng2 LacZ* KI mice. The *in situ* hybridization data of *Ntng1* and *Ntng2* genes in P28 mice were quoted from Allen Brain Atlas [62] [ http://www.brain-map.org/]. (−), no expression; (+), weak expression; (++), moderate expression; (+++), strong expression; (n.d.), no data. Click here for file
