# Supplementary material for: Identification of transcriptional regulatory elements for Ntng1 and Ntng2 genes in mice
Source: Mol Brain. 2014 Mar 19;7:19. doi: 10.1186/1756-6606-7-19 (PMC4000137; doi:10.1186/1756-6606-7-19)
Supplement: Additional file 2: Figure S1 — LacZ-stained brain sections of Ntng1 and Ntng2 LacZ KI mice at high magnification. (A-E) X-gal staining of Ntng1-LacZ KI mice. (F-J) X-gal staining of Ntng2-LacZ KI mice. (A-C, E-H, J) Coronal sections (25 μm) at P21. (D, I) Horizontal sections (25 μm) at P21. Sections were lightly counterstained with hematoxylin. In the anterior olfactory nucleus, Ntng1 was expressed in the neurons of dorsal-, lateral-, medial-part, and Ntng2 was expressed in the neurons of the external part. In the piriform cortex, Ntng1 was expressed in the semilunar neurons of layer II, whereas Ntng2 was expressed in the pyramidal and polymorphic neurons of layer III. In the cerebral cortex, Ntng1 was expressed in layer V neurons, whereas Ntng2 was expressed in neurons in layers II/III, IV, and VI. In the entorhinal area, Ntng1 was expressed in layer II of the lateral entorhinal area (LEC) and layer III throughout the entorhinal area, and Ntng2 was expressed in layer II of the medial entorhinal area (MEC) and layer III of the LEC. In the amygdala, Ntng1 was expressed in the lateral amygdala nucleus (LA), whereas Ntng2 was expressed in the basolateral amygdala nucleus (BLA). Scale bar: 1.0 mm for all panels. [file 1756-6606-7-19-S2.pdf]

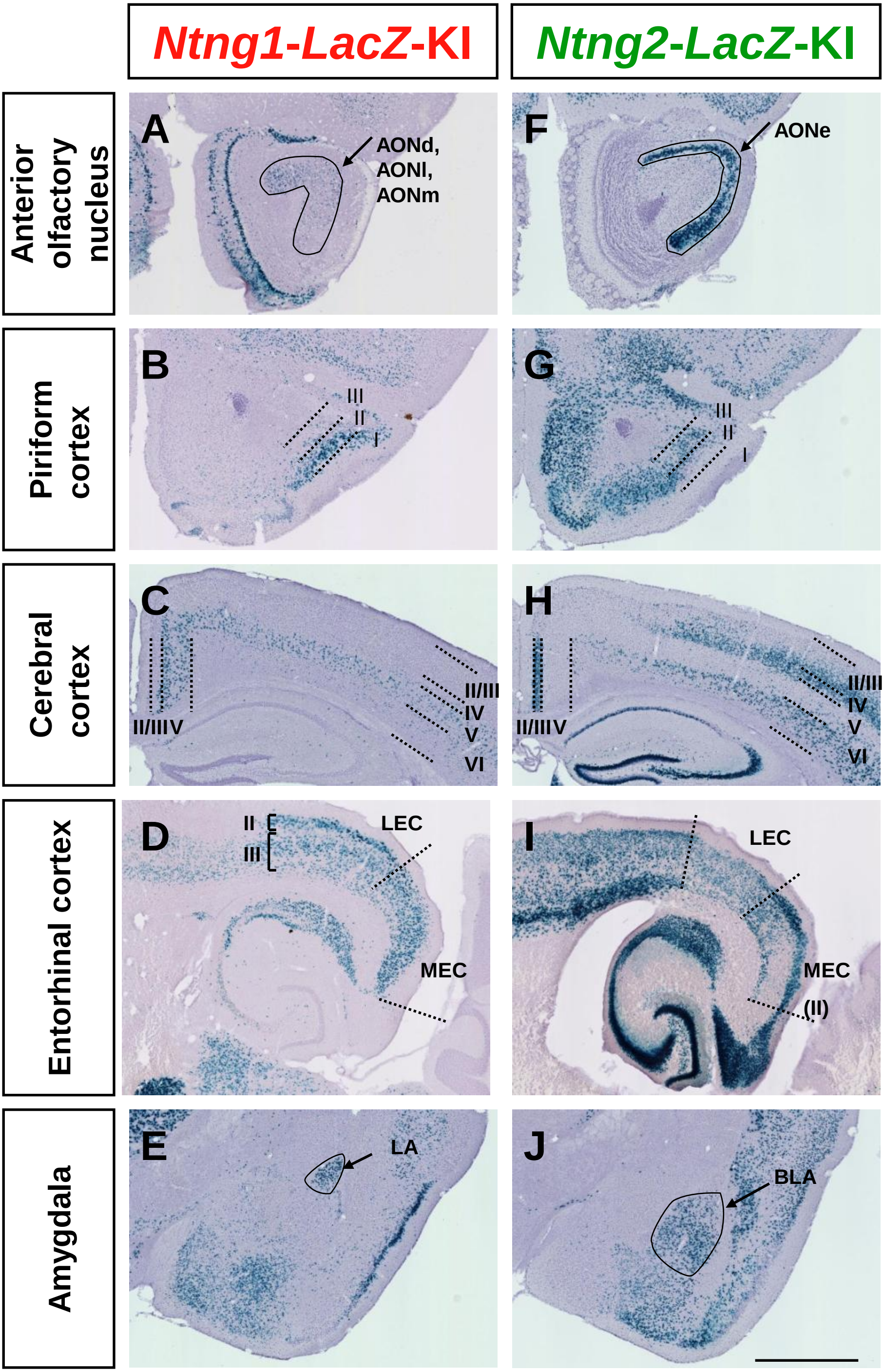

Ntng1-LacZ-KI
Ntng2-LacZ-KI
Anterior olfactory nucleus
Piriform cortex
Cerebral cortex
Entorhinal cortex
Amygdala
A
AONd, AONl, AONm
F
AONe
B
III
II
I
G
III
II
I
C
II/III
IV
V
VI
II/III V
H
II/III
IV
V
VI
II/III V
D
II
III
LEC
MEC
I
LEC
MEC
(II)
E
LA
J
BLA
