# Supplementary figures and images for: Identification of transcriptional regulatory elements for Ntng1 and Ntng2 genes in mice
Source: Mol Brain. 2014 Mar 19;7:19. doi: 10.1186/1756-6606-7-19 (PMC4000137; doi:10.1186/1756-6606-7-19)

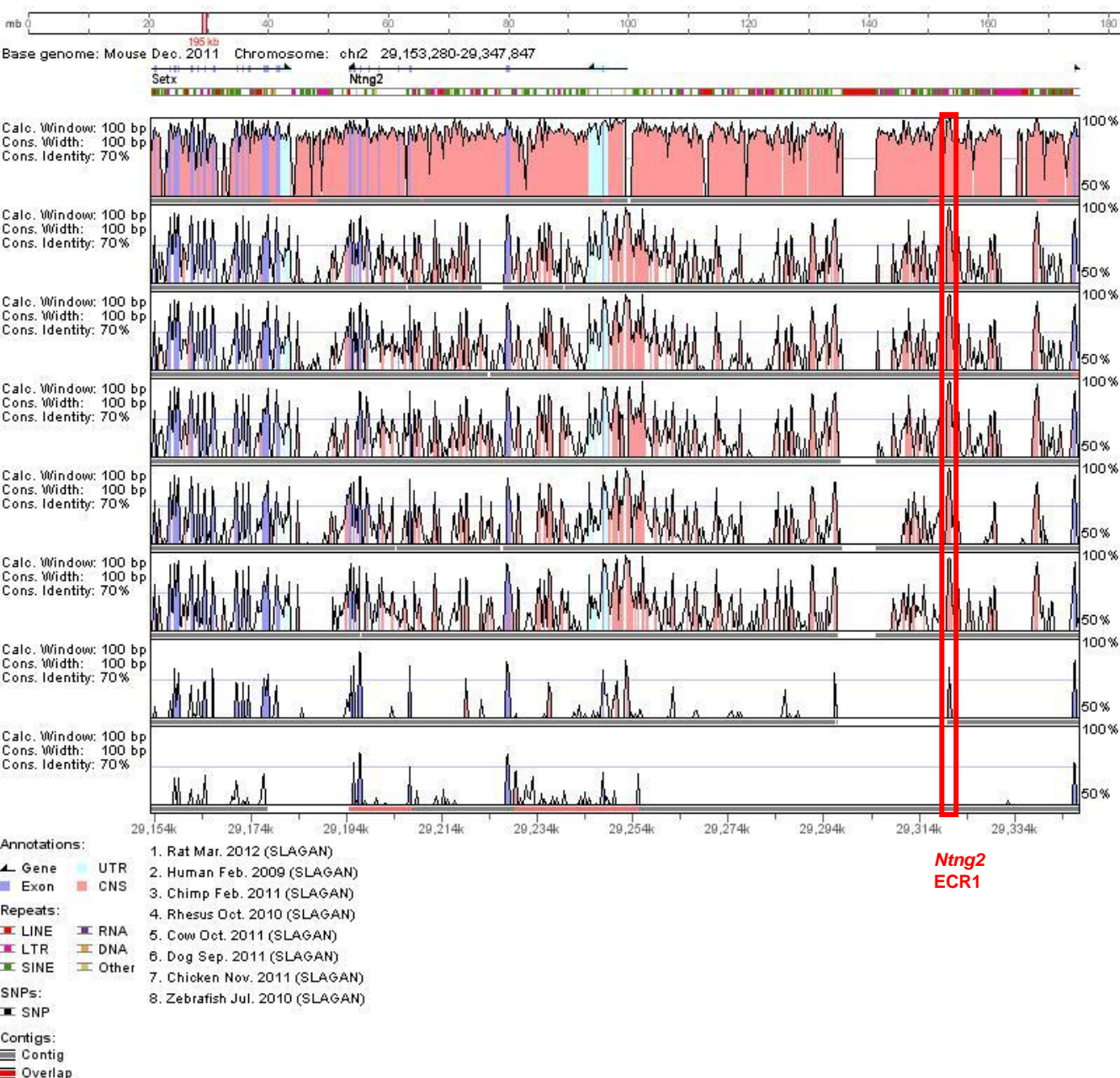

Supplement: Additional file 4: Figure S2 — Evolutionarily conserved region (ECR) in the mouse Ntng2 locus. Analysis of the Ntng2 locus covered by the Ntng2-BAC (mouse Dec. 2011 [GRCm38/mm10] assembly; chr2: 29,153,280-29,347,847). Comparative genomic analysis of the mouse Ntng2-BAC sequence by the VISTA genome browser ( http://genome.lbl.gov/vista/index.shtml). Percent nucleotide identities between mouse and other species (rat, human, chimp, rhesus, cow, dog, chicken, and zebrafish; from top to bottom) are plotted as a function of the position along the mouse sequence. Peaks of evolutionarily conserved overlapping exons of Ntng2 and neighboring genes are shaded blue. Aligned regions with more than 70% identity over 100 bases are shaded pink. Ntng2 ECR1, indicated by the red rectangle, represents the most highly conserved region and locates within segment II (Figures 3 and 5). [file 1756-6606-7-19-S4.pdf]

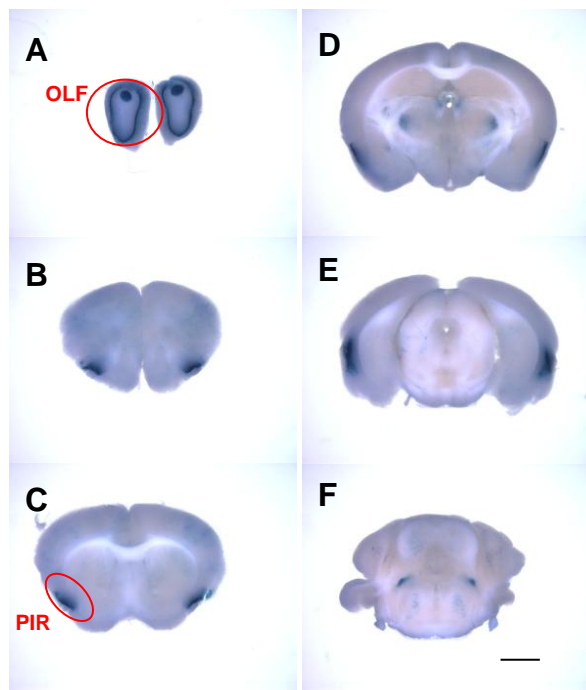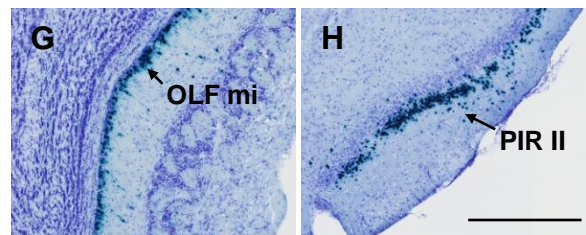

Supplement: Additional file 10: Figure S6 — Recombination patterns in Ntng1-Del II-Cre : Rosa-NLSLacZ mice. (A-H) LacZ activies (blue signals) of Ntng1-Del II-Cre :Rosa-NLSLacZ [44] mice. Coronal sections (A-F: 400 μm, G-H: 25 μm) were stained with X-gal solution. Sections were lightly counterstained with hematoxylin. Red circles indicated the region-specific LacZ activities. In the olfactory areas, Cre was highly expressed in the olfactory bulb mitral cells. In the piriform cortex, Cre was expressed in the semilunar neurons of layer II. Scale bar: 2.0 mm for A-F panels, 500 μm for G-H panels. [file 1756-6606-7-19-S10.pdf]
