# Supplementary material for: Identification of transcriptional regulatory elements for Ntng1 and Ntng2 genes in mice
Source: Mol Brain. 2014 Mar 19;7:19. doi: 10.1186/1756-6606-7-19 (PMC4000137; doi:10.1186/1756-6606-7-19)
Supplement: Additional file 6: Figure S4 — Multiple sequence alignment and candidate transcription-factor binding sites in Ntng2-ECR1. Sequence comparisons of the Ntng2 enhancer Ntng2-ECR1 sites between mouse, rat, human, chimp, rhesus, cow, dog, and chicken species. Putative transcription factor binding sites are highlighted in the differential colors, respectively. Seventeen potential highly conserved binding sites were identified (searched results are from DiAlign TF). [file 1756-6606-7-19-S6.pdf]

V$CEBP V$MEF2 V$AP1R O$VTBP V$ABDB V$FKHD V$FAST V$MYOD V$HOMF V$CART V$MYBL V$KLFS V$LHXF V$CREB V$PARF V$OVOL V$NR2F

```
alignment
position     1201...... 1211...... 1221...... 1231...... 1241...... 1251...... 1261...... 1271...... 1281...... 1291......
Mouse   1022 -----TATCT AATCTGTTCT GAAGAACAAC CATTTTGAAA TTGGGCAAAA ATACTGCCTA ATATATTTAA ACATATATAT TTTTATTTTT GTATCATTTT
Rat      949 -----TATCT AATCTGTTCT GAAGAACAAC CATTTTGAAA TTGGGCAAAA ATACTGCCTA ATATAGCTAA ACATATATAT TTTTATTTTc GTATCATTTT
Human    323 ------ATCT AATCTGTTCT GGAGAACAAC CATTTTGAAA TTGGGCAAAA ATATAGCCTA ATATATTTAA ACATATATAT TTTTATTTTT ATATAA-TTT
Chimp    317 ------ATCT AATCTGTTCT GGAGAACAAC CATTTTGAAA TTGGGCAAAA ATATAGCCTA ATATATTTAA ACATATATAT TTTTATTTTT ATATAA-TTT
Rhesus   303 ------ATCT AATCTGTTCT GGAGAACAAC CATTTTGAAA TTGGGCAAAA ATATAGCCTA ATATATTTAA ACATATATAT TTTTATTTTT ATATAA-TTT
Cow      288 ttttaTATCT AATCTGTTCT TGAGAACAAC CATTTTGAAA TTGGGCAAAA ATACAGCCTA ATATATTTAA ACATATATAT TTTTATTTTT ATATAA-TTT
Dog      244 -----TATCT AATCTGTTCT TGAGAACAAC CATTTTGAAA TTGGGCAAAA ATACAGCCTA ATATATTTAA ACATATATAT TTTTATTTTT ATATAA-TTT
Chicken   17 ------ATTT AACCTGCTTT GGATAGTTGT CCTTTAGAAA GTGTGCAAAA tag------A TTAAATTTTA ACtgc----- ---------- --ATCA-CTT
                   ** * ** *** * *   * *      * *** ****  ** ******          *  ** *  * * **                    ** *  **

alignment
position     1301...... 1311...... 1321...... 1331...... 1341...... 1351...... 1361...... 1371...... 1381...... 1391......
Mouse   1117 AAAAATAAAC CCATGGATGT ACATTTTCCA AGcGCCTGCC TGCTGGATTC CTAATGTAAT AC-------A ACATGACTCA GAGCTAAATT TAAAGAGAGA
Rat     1044 AAAAATAAAC GCATGGATGT ACATTTTCCA AGc-CCTGCC TGCTGGATCC CTAATGTGAT GC-------A ACATGACTCA GAGCTAAATT TAAAGAGAGA
Human    416 AAAAATAAAC GCATTGATGT ACATTTTCCA AGTGCCTGCC TGCTGAATTC CTAATGTAAT AC-------A ACATGACTCA GAGCTAAATT TAAAGAGAGA
Chimp    410 AAAAATAAAC GCATTGATGT ACATTTTCCA AGTGCCTGCC TGCTGAATTC CTAATGTAAT AC-------A ACATGACTCA GAGCTAAATT TAAAGAGAGA
Rhesus   396 AAAAATAAAC GCATTGATGT ACATTTTCCA AGTGCCTGCC TGCTGAATTC CTAATGTAAT AC-------A ACATGACTCA GAGCTAAATT TAAAGAGAGA
Cow      387 AAAAATAAAC GCATTGATGT ACATTTTCCA AGTGCCTGCC TGCTGAATTC CTAATGTAAT AC-------A ACATGACTCA GAGCTAAATT TAAAGAGAGA
Dog      338 AAAAATAAAC GCATTGATGT ACATTTTCCA AGTGCCTGCC TGCTGAATTC CTAATGTAAT AC-------A ACATGACTCA GAGCTAAATT TAAAGAGAGA
Chicken   87 AAAAATAcat gtgcagatct ctgaagtga- ---------- --CTGGGATC CGAATGTAta gggcagcagA ACATGACTCA AAGCCCAATT TAAAGACAGA
             *******                                  ***    * * *****            * **********  ***  **** ****** ***

alignment
position     1401...... 1411...... 1421...... 1431...... 1441...... 1451...... 1461...... 1471...... 1481...... 1491......
Mouse   1210 AGCACATATG TTTAACGCAG GCACCAACTT TCAATCTGGG GAGAA-CGGA GTCAAAGGGA ------TTTT TTTTTAGCGT ACGAGTGGAC CCAGTGCAAA
Rat     1136 AGCACATATG TTTAACGCAG GCACCGGCTT TCAATCTGGG GAGAAACGGA GTCAAAGGGA -------TTT TTTTTAGCGT ACGAGTGGAC CCAGTGCAAA
Human    509 AGCACATATG TTTAAAGCAG GCACCAACTT TCAATCTGAG GAGAAACAGA GTCAAAGtat ------TT-T TTTTTAGCGT GTGAGTGGAC TGAGTGCAAA
Chimp    503 AGCACATATG TTTAAAGCAG GCACCAACTT TCAATCTGAG GAGAAACAGA GTCAA-AGTA ------TTTT TTTTTAGCGT GTGAGTGGAC TGAGTGCAAA
Rhesus   489 AGCACATATG TTTAAAGCAG GCACCAACTT TCAATCTGAG GAGAAACAGA GTCAAAAGTA ------TTTT TTTTTAGCGT GTGAGTGGAC TGAGTGCAAA
Cow      480 AGCACATATG TTTAAAGCAG GCACCAACTT TCAATCTGAG GAGAAACAGA GTCAAAAGTA ------TT-T TTTTTAGCGT GTGAACGGAC TGAGTGCAAA
Dog      431 AGCACATATG TTTAAAGCAG GCACCAACTT TCAATCTGAG GAGAAACAGA GTCAAAAGTA ------TT-T TTTTTAGCGA GTGAACGGAC TGAGTGCAAA
Chicken  174 A----ATATG TTTTGAGCAA GCATGAACAG TTAGACTGAA CAGCA----A GGCAAAAGca tgggttTTTT TTTTTAGCTG TTGAGTGGAC TAAATGCAAT
             *    ***** ***   ***  ***    *  * *  ***   ** *    ** ***              * * ********  **  ****   * *****

alignment
position     1501...... 1511...... 1521...... 1531...... 1541...... 1551...... 1561...... 1571...... 1581...... 1591......
Mouse   1303 GTGAATTA-T CTCCAAGCCA TCTGGGAAGT CTGGTGTTTT TCAATGCCTT CACGACTCTA ATTTTAAGCC GTTATTTGGC CTTTAAATAA CAATATCTTT
Rat     1229 GTGAATTA-T CTCCAAGCCA TCTGGGAAGT CTGGTGTTTT TCAATGCCTT CACGACTCTA ATTTTAAGCC GTTATTTGGC CTTTAAATAA CAATATCTTT
Human    602 GTGAATTA-T CTCCAAGCCA TCTGGGAAGT CTGGTGTTTT TCAATGCCTT CACAACTCTA ATTTTAAGCC GTTATTTGGC CTTTAAATAA CAATATCTTT
Chimp    596 GTGAATTA-T CTCCAAGCCA TCTGGGAAGT CTGGTGTTTT TCAATGCCTT CACAACTCTA ATTTTAAGCC GTTATTTGGC CTTTAAATAA CAATATCTTT
Rhesus   583 GTGAATTA-T CTCCAAGCCA TCTGGGAAGT CTGGTGTTTT TCAATGCCTT CACAACTCTA ATTTTAAGCC GTTATTTGGC CTTTAAATAA CAATATCTTT
Cow      573 GTGAATTA-T CTCCAAGCCA TCTGGGAAGT CTGGTGTTTT TCAATGCCTT CACAACTCTA ATTTTAAGCC GTTATTTGGC CTTTAAATAA CAATATCTTT
Dog      524 GTGAATTA-T CTGAGAGCCA TCTGGGAAGT CTGCTGTTTC TCAAT-CCTT CGCAACTCTA ATTTTAAGCC GTTATTTGGC CTTTAAATAA CAATGTCTTT
Chicken  266 GTGAATTtgT CTGCAAACCA TCTGTGATTT CTGGTATTTT TTGATGCCTT CACAGCTCTA ATTTTAAGCA GTTACGTAGC CTTTAAATAA CGATAg--TT
             *******  * **   * *** ****  **  * *** * ***  *  ** **** * *  ***** *********  ****  *  ** ********** * **    **

alignment
position     1601...... 1611...... 1621...... 1631...... 1641...... 1651...... 1661...... 1671...... 1681...... 1691......
Mouse   1402 TCTTTCCATT GGCTTTTGCA CCAGATTCCG TATTTATTGC GATATTGACT CCAGAAAGAG ACCTATC--T TTGGGAGCAA AAAAGAGCAA AGTTATTGAA
Rat     1328 TCTTTCCATT GGCTTTTGCA CCAGATTCCG TATTTATTGC GATATTGACT CCAGAAAGAG ACCTATC--T TTGGGAGCAA AAAAGAGCAA AGTTATTGAA
Human    701 TCTTTCCATT GGAATTTGCA CCAGAT-CCG CATTTATTGC GCTATCGACT CCAAAAAGAG ACCTATC--T TTGAGAGCAA AAAGAGCAAA AGTTATTGAA
Chimp    695 TCTTTCCATT GGAATTTGCA CCAGAT-CCG CATTTATTGC GCTATCGACT CCAAAAAGAG ACCTATC--T TTGAGAGCAA AAAGAGCAAA AGTTATTGAA
Rhesus   682 TCTTTCCATT GGAATTTGCA CCACAT-CCG CATTTATTGC GCTATCGACT CCAAAAAGA- -CCTGTC--T TTGAGAGCGA AAAGAGCAAA AGTTATTGAA
Cow      672 TCTTTCCATT GGAATTTGCA CCAGATTCCG CATTTATTGC ACTATCGACT CCAAAAAGAG ACCTATCttT TTGAGAGCAA AAAGAGCAAA AGTTATTGAA
Dog      622 TCTTTCCATT GGAATTTGCA CCAGATTCCG CATTTATTGC GCTATCAGCT CCAAAAAGAG ACCTATC--T TTGAGAGCAA AAAGAGCAAA AGTTATTGAA
Chicken  364 TCTTCCCATT CGGTTTTACA TCAGATTCCA CACTTg---- ---------- ---------- ---------- ---------- ---------- ----------
             **** *****  *  *** ** ** ** **  * **

alignment
position     1701...... 1711...... 1721...... 1731...... 1741...... 1751...... 1761...... 1771...... 1781...... 1791......
Mouse   1500 CACATTACTC TGCAGATTGG ---------- ---CTCTGGA GTtggggGGG GGGAACAGCA GTCTGATA-T TTTGCAAATG AAGTTTGACA TATTCTGGGA
Rat     1426 CACATTACTC TGCAGATTGG ---------- ---CTCTGGA GT-----GGG GGGAACAGCA GTCTGATA-T TTTGCAAATG AAGTTTGACA TATTCTGGGA
Human    798 CGTATTACTC TGCAGATTGG ---------- ---CTCTGGA -AAAAA---- ---AACAGCA GTCTGATA-T TTTGCAAATG AAGTTTGACA TATTCTGGGA
Chimp    792 CGTATTACTC TGCAGATTGG ---------- ---CTCTGGA -AAAAA---- ---AACAGCA GTCTGATA-T TTTGCAAATG AAGTTTGACA TATTCTGGGA
Rhesus   777 CGTATTACTC TGCAGATTGG ---------- ---CTCTGGG GAAAAA---- ---AACAGCA GTCTGATA-T TTTGCAAATG AAGTTTGACA TATTCTGGGA
Cow      772 CGTATTACTC TGCAGATTGG ---------- ---CTCT-GG GAAAAA---- ---AACAGCA GTCTGATA-T TTTGCAAATG AAGTTTGACA TATTCTGGGA
Dog      720 CGTATTACTC TGCAGATTGG ---------- ---CTCT-GG GAAAAA---- ---AACAGCA GTCTGATA-T TTTGCAAATG AAGTTTGACA TATTCTGGGA
Chicken  400 ---ATCACTT TGAAGAagtg gctgagtata ttcCTCTGCA GAATAA---- ---AACAGCA GctttttatT TTTACAAGTG AAATGTGACA CATTTTGGGC
                ** ***  ** ***                ****                ******* *         * *** *** ** ** * *****  *** ****
```
