# Supplementary material for: Identification of transcriptional regulatory elements for Ntng1 and Ntng2 genes in mice
Source: Mol Brain. 2014 Mar 19;7:19. doi: 10.1186/1756-6606-7-19 (PMC4000137; doi:10.1186/1756-6606-7-19)
Supplement: Additional file 8: Figure S3 — ECR in the mouse Ntng1 locus. Analysis of the Ntng1 locus covered by the Ntng1-BAC (mouse Dec. 2011 [GRCm38/mm10] assembly; chr3:110,101,082 -110,318,433) by the VISTA genome browser ( http://genome.lbl.gov/vista/index.shtml). Percent nucleotide identities between mouse and other species (rat, human, chimp, rhesus, cow, dog, chicken, and zebrafish; from top to bottom) are plotted as a function of the position along the mouse sequence. Peaks of evolutionary conservation overlapping exons of Ntng1 and neighboring genes are shaded blue. Aligned regions with more than 70% identity over 100 bases are shaded pink. Ntng1 ECR1 and ECR2, indicated by the red rectangles, represent one of the most highly conserved regions and locate within segments II and VI, respectively (Figures 5 and 6). [file 1756-6606-7-19-S8.pdf]

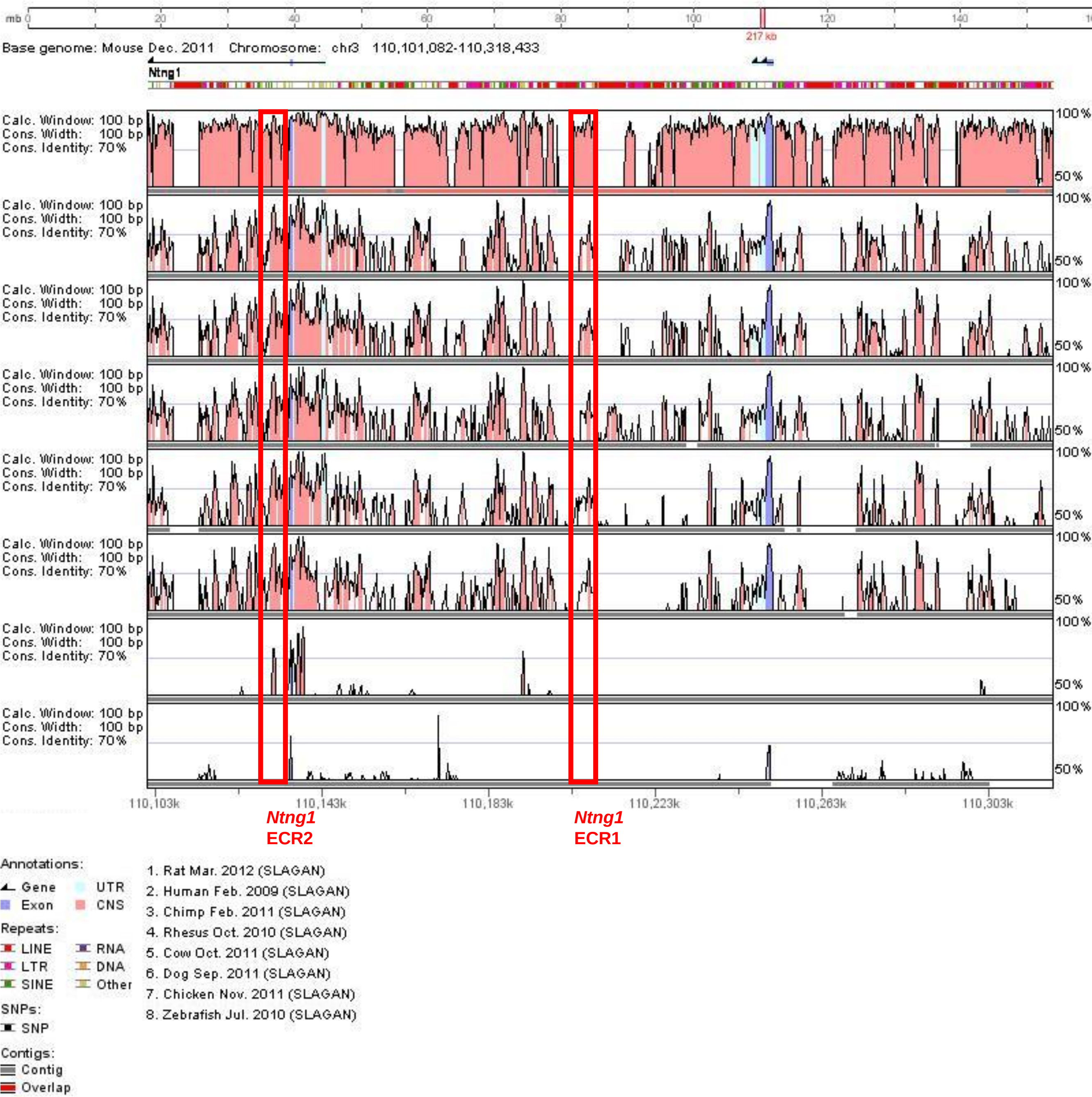
Base genome: Mouse Dec. 2011 Chromosome: chr3 110,101,082-110,318,433
217 kb
Ntng1
Calc. Window: 100 bp
Cons. Width: 100 bp
Cons. Identity: 70%
100%
50%
110,103k
110,143k
110,183k
110,223k
110,263k
110,303k
Ntng1 ECR2
Ntng1 ECR1
Annotations:
Gene
UTR
Exon
CNS
Repeats:
LINE
RNA
LTR
DNA
SINE
Other
SNPs:
SNP
Contigs:
Contig
Overlap
1. Rat Mar. 2012 (SLAGAN)
2. Human Feb. 2009 (SLAGAN)
3. Chimp Feb. 2011 (SLAGAN)
4. Rhesus Oct. 2010 (SLAGAN)
5. Cow Oct. 2011 (SLAGAN)
6. Dog Sep. 2011 (SLAGAN)
7. Chicken Nov. 2011 (SLAGAN)
8. Zebrafish Jul. 2010 (SLAGAN)
