# Supplementary material for: Identification of transcriptional regulatory elements for Ntng1 and Ntng2 genes in mice
Source: Mol Brain. 2014 Mar 19;7:19. doi: 10.1186/1756-6606-7-19 (PMC4000137; doi:10.1186/1756-6606-7-19)
Supplement: Additional file 9: Figure S5 — Multiple sequence alignment and candidate transcription-factor binding sites in Ntng1-ECR1. Sequence comparisons of the Ntng1 enhancer Ntng1-ECR1 sites between mouse, rat, human, chimp, rhesus, cow, and dog species. Putative transcription factor binding sites are highlighted in the differential colors, respectively . Eight potential highly conserved binding sites were identified (searched results are from DiAlign TF). [file 1756-6606-7-19-S9.pdf]

V$CART V$BRNF V$SORY V$NKXH V$LEFF V$HOMF V$ABDB V$CDXF

```
alignment
position    1401...... 1411...... 1421...... 1431...... 1441...... 1451...... 1461...... 1471...... 1481...... 1491......
Mouse   840 ATTTTAAAGT -----TT--- AAAATGTAAG GAGCTGAAAA A-TATGATGG TTA------- ---TTATTCA ATTTTCAATA TGTGTAATTT AACTTGATTA
Rat     826 ATTTTAAAGT -----CT--- AAAATGTAAG GAGCCGAAAA ACTATGATTG TTA------- ---TTATTCA ATTTTCAATG TGTATAATAT AACTTGATTA
Human  1167 GTTATAAAGT -----CT--- GAAATATAAG AAAGTTTAAA AAGCAGC--- -TA------- ---TTAATTA ACTTTCAGTC TGTGTGATAT AATTTGATTC
Chimp  1236 GTTATAAAGT -----CT--- GAAATATAAG AAAGTTTAAA AAGCAGC--- -T-------- ------ATTA ATTTTCAGTC TGTGTGATAT AATTTGATTC
Rhesus 1174 GTTACACAGT -----CT--- GAAATATAAG AAAGTTTAAA AAGCA-C--- -TA------- ---TTAATTA ATTTTCAGTC TGTGTGGTAT AATTTTATTC
Cow     840 CTTTTATAat gtctcTT--- AATATATAAG AAATTTAAAA TATAGGAgcc atagtagcca tacTAAATTA ATTTTCAGTC TATGTGGCCT AATTTAATTC
Dog     916 GTTATAAAGT -----TTctg AATATATAAG AAATGAAAAG AAATAAC--- -GA------- ---TTACTCA ATTTTCAATC TATGTCATCT AATTCAGTTC
             **  * *         *     * ** ****  *     **                              * * ***** *  * * *    * ** *   ** 

alignment
position    1501...... 1511...... 1521...... 1531...... 1541...... 1551...... 1561...... 1571...... 1581...... 1591......
Mouse   921 ----CAAACA CAATGTGTGC TGAATACAGA GTA-AT-AAA TCCACTTATT AA----TTTG TATA-GTTTG TTTCAATGTA CTACATACAT AAATATTCAT
Rat     908 ----CAAACA TAATGTGT-- ---------- GTA-AT-AAA TCCCCTTATT AA----TTTG TATG-CTTTG TTTCAATGTA CTACATAAAT AAAAATTTAT
Human  1245 TTTCCAAATA CTATGCCTGA TGAACACAAG TAATAT-AAG GCTTCTCATT CA----TTTG TGTATCTTTG CTTTAATGTT CTTAATAAAT AAGGTTTCTT
Chimp  1310 TTTCCAAATA CTATGCCTGC TGAATACAAG TAATAT-AAG GCTTCTCATT CA----TTTG TGTATCTTTG CTTTAATGTT CTTAATAAAT AAGGTTTCTT
Rhesus 1251 TTTTCAAATA CTATGCATGT GGAATACAAG TAACAT-AAA TCTTCTCATT CA----TTTG TGTATCTGTG CTTTAATGTT CTTAATAAAT AAGATTTCTT
Cow     937 TTTTCACATA TTATGCATGA TGAATAAAAA GTA-ATAAAA ATTTCTGATT CC----TTTG TATAATTTTt gcttcaatgt cc-----AAT AAGGCTTCTT
Dog     997 TTTCCAAATA Ccac-CATGC TGAACACAAA GTA-ATAAAA Aagtttctgt ttgcttTGTG TATT-TTTTG CCTCAAAGTC CTTCATAAAT TAGGCTTCTT
                ** * *        *               * ** **                  * ** * *   * *                    **  *   **  *

alignment
position    1601...... 1611...... 1621...... 1631...... 1641...... 1651...... 1661...... 1671...... 1681...... 1691......
Mouse  1010 GAGAATCAAA TGACTATAGG TTTCAGA-TT TtTCCCTCCG CATGAATACA ATCAAGTAAA ATTCTCTATG CGTTGGccga tgTTTCCACA GTTCCTGGTA
Rat     985 GAAAATCAAA TCACTATAGG TTTCAGAtTT T-TCACTCCT CGTGTATACA CTAAAGTAAT ATTCTCTATT CATTGGAGAG CATTTCCACA GTTCCTGGCA
Human  1340 AAAAATGAAA TGGCAACAGA TTTCTGA-TT T-TCACTCTT CATAAAAATC ATATAGTAAT ATCCTCTTCT TATTTGAAAT CTTGACTACT CTTCCTGACA
Chimp  1405 AAAAATGAAA TGGCAACAGA TTTCTGA-TT T-TCACTCTT CATAAAAATC ATATAGTAAT ATCCTCTTCT TATTTGAAAT CTTGACTACT GTTCCTGACA
Rhesus 1346 GAAAATGAAA TGGCAACAGG TTTCTGA-TT T-TCACTCTT CATAAAAATC ATATAGTAAT ATCCTCTTCT TATTTGAAAT CTTGACTACT GTTCCTGACA
Cow    1027 AAGAACCAAA TGGCAATAGG TTTTAGA-TT T-CCACTCTT CATGAAATTA GTAAACTAAC ATTTTTTTTC TATTTAAAAT CTTCACTTTT ATTACTGGCA
Dog    1094 AAAAACCAAC TAGCAACCGG TGTGAGA-TT T-TTACTCTT CATGAAAATA GTCAAGTAAT TATCTCTTTT TATTTGAAAT CTTTACTCCT ATTCCTGGCA
             * **  ** *  * *  * * *  ** ** *    ***   * *  *     *  * ***      * *       **          *  *      ** ***  *

alignment
position    1701...... 1711...... 1721...... 1731...... 1741...... 1751...... 1761...... 1771...... 1781...... 1791......
Mouse  1109 CTGGTTAGTT CTAAGGTACT ATTTTCTACC TTTAATAAAA CACTAAAGCA ACTCTTCCAA AAGGCTCACC TGGTATAATG AGAAATGTAT TTGAATGACC
Rat    1084 CTGGTTATTT CTAAGGTCCA ATTTTCTACT TGTAATAAAA CACTAAAGCA ACTCTTCCAA AAAGCTTACT TGATCTAATG AGAAATGTAT TTGAATGACC
Human  1438 TTTGCTAGTT CTAGTGTACT CTTTGCTACT TGTTATAAAA TGTAAAAACA ACATTTCTGA AAGGCCCACT CAATGTGATA AAGAATCTAT TTGAATGGCT
Chimp  1503 TTTGCTAGTT CTAGTGTACT CTTTGCTACT TGTTATAAAA TGTAAAAACA ACATTTCTGA AAGGCCCACT CAATGTGATA AAGAATCTAT TTGAATGGCT
Rhesus 1444 TTTGCTGGTT CTAGTGTACT CTTTGCTACT TGTTATAAAA TGTAAAAACA ACATTTCTGA AAGGCCCACT CAATGTGATA AAGAATCTAT TTCAATGACT
Cow    1125 TTTGCTA-TT CTAGGGTACT ATTTGCTATT TGTTAc--AA TGTAAAAGCA ACAATTCTGA AAGCTTCACT CAGTATGATG AAGAATGTAT TTCAATGACT
Dog    1192 TTTCCTGGTT CTAGTGTACT ATTTGCTATT TGCTATAAAA TGCAAAAGCA ACATATCTGA ACGGCTCACT CAATATGATG AAGAATGTAT TTGAATGACT
             *   *  ** ***  ** *  *** ***  *   *   **     *** ** **   **  * *      **     * * ** *  *** *** ** **** * 

alignment
position    1801...... 1811...... 1821...... 1831...... 1841...... 1851...... 1861...... 1871...... 1881...... 1891......
Mouse  1209 CAAAATACAC TTATTCCATT TAACCAAAGT GCATATACAC TTTGATATTA TTTGTTTTAA TTTTTCAATG TGTCTgg--T ATAGTCAACC AATTTGAGCT
Rat    1184 CAAAATACAC TTATTCCATT TAACCAAAGT GCATATACAT TT-GATATTA TTTGTTTTAA TTTTTCAATA TGTCTc--AT ATAGCCAACC AATTTGAGCT
Human  1538 CAAAAGACAC TTATTCTATT TAACCAATGT CTGTATGCAT TTTGATATTA TTTTACTTTA ATTTTAAAAA TGTACATTAT TTATCCAACC AGTTGGAACT
Chimp  1603 CAAAAGACAC TTATTCTATT TAACCAAAGT CTGTATGCAT TTTGATATTA TTTTACTTTA ATTTTAAAAG TGTACATTAT TTATCCAACC AGTTGGAACT
Rhesus 1544 CAAAAGACAC TTATTCTATT TAACCAAAGT CTGTATGCAT TTTGATATTA TTTTACTTTA ATTTTTTAAA TGTACATTAT TTATCCAACC AATTGGAACT
Cow    1222 CAAAATACAC TTACTCTATT TAATCAATGT GTATATGCAT TTTGATATCA TTTTA----- ATTTTTTAAG TGTACATTAC TTATCCAACC AATTGGAACT
Dog    1292 CAAAGGACAC TTACTCTATT TAACCAAAGT GTGTATGCAT TTTGATATTA TTTTATTTTA ATTTTTTAAA TGTACATTAC TTATCTAGCC AATTGGAACT
            ****  **** *** ** *** *** *** **    *** **  ** ***** * ***         ****  *   ***         **   * ** * ** ** **

alignment
position    1901...... 1911...... 1921...... 1931...... 1941...... 1951...... 1961...... 1971...... 1981...... 1991......
Mouse  1307 ATTCCAGGAT GCGTTCACTT ATAAATGCTT TTACAATTCC AAGTGGCTCA TAATAGCGTA TTCGGAACTA TGCAAGCTGA TACTCAAAGT GGATGTTAAG
Rat    1281 ATTCTAGGAT GCATTCACTT ATGAATGCTT TTACAATTCC AAATGGCTCA TAATAGTATA TTTGAAACTA TGAGAGCTGA TACTCTAAGT GGATGTTAAG
Human  1638 GTTCTGGTTT GCATTCATTT ATGAATGCTT TTAAAATTCC AAATGGCTCA TAA--GCATA TTTGAAACTA CTGGAGTTGA TGCTCTAAGT GAATGTTAAG
Chimp  1703 GTTCTGGTTT GCATTCATTT ATGAATGCTT TTAAAATTCC AAATGGCTCA TAA--GCATA TTTGAAACTA CTGGAGTTGA TGATCTAAGT GAATGTGAAG
Rhesus 1644 GTCCTGGTAT GCATTCATTT ATGAATGCTT TTAAAATTCC AAATGGCTCA TAA--GCATA TTTGAAAGTA CTGGAGTTGA TGTTCTAAGT GAATGTTAAG
Cow    1317 c-TCTACCAT GCATTTATTT ATGAATGCTT TTAAAATTCC AAATGGCTCA TAATGGCATA TTTGAAACTA CTAGAGTTAA TGCTGTAAGT GAATGCTAAT
Dog    1392 ATTTTAGAAT GCATTTATTT ATGAATGCTT TTAAAATTCC AAATGGCTCA TAATCGCATT TCTGAAGCTA CTAGAGTTGA TGC------T GAGTATTAAG
                     * ** ** * ** ** ******* *** ****** ** ******* ***  *  * *  * *  **     ** * * *        * *  *   ** 

alignment
position    2001...... 2011...... 2021...... 2031...... 2041...... 2051...... 2061...... 2071...... 2081...... 2091......
Mouse  1407 ACTCATAATA CAAATGAAAA GCTGTTACTG CATGAAACTC AAGT-CATTT CCTAGAGCAT TATACCCCAG CAATTTAAAA GACTTAC--T CAAAACATGA
Rat    1381 ACTCATAATA CAAATGAAAA GCTGTTACTG CACAAAACTC AAGTC-ATTT CCCAGAGCAT AATATCCCAG CAATTTAAAA GACTTAC--T CAAAACACGA
Human  1736 ACTCATAACC CAAATGAAAA GCTGTTATTG TACAACACTC AAGTCCATTT TCTAGGGCAT AATATCTCAG CAGTTTTTAA CACTTCTATT CAAAATACCA
Chimp  1801 ACTCATAACC CAAATGAAAA GCTGTTATTG TACAACACTC AAGTCCATTT TCTAGGGCAT AATATCTCAG CAGTTTTAAA CACTTCTATT CAAAATACCA
Rhesus 1742 ACTCATAACC CAAATGAAAA GCTGTTATTG TACAACACTC AAGTCCATTT TCTAGGGCAT AATATCTCAG CAATTTTAAA CACTTCTATT CAAAATACCA
Cow    1416 ACTCATAACC CAAATGAAAA GCTGCTATTG CAGAACACTC AAGTC-AcTT TCTAGGGTGT AATATCACAG CAAATTTAAA AACATtttTT CAAAATACCA
Dog    1486 ACTCCTAACG CAAATAAAAA GCTGTTATTG CAGACCACTC AAGCCCATTT TCTAGAGCAT AATATCTCAG CAATTTTAAA GACGGTTATT CAAAATACCC
            **** ***   ***** **** **** ** **  *    **** ***   * **  * ** *  *  *** * *** **  **  **   **      * ***** *   

alignment
position    2101...... 2111...... 2121...... 2131...... 2141...... 2151...... 2161...... 2171...... 2181...... 2191......
Mouse  1504 AGAGAAATTA GAAAATGTGG TGACAACTCC TATGCAAACT TAGATTGCTG -AAATCATTT TGAAATTAAC CTTCTGGAAA AGATCAGCCA ACTAAACACA
Rat    1478 AGAGAAATTA GACAATATGG TGACAACTCC TATGCAAACT TAGATTGCTG -AAATCATTT TGAAATTAAC CTTCTGGAAA AGATCAGCCA ACTAAACATG
Human  1836 AGAAGAATTG GACAATATGA TGGCAATCCC TTTGCAAACT TCAGCTGTTA AAAATCATTC TGAAAATATC CTCCTGGAAA ATATCAACCA C----ACTTA
Chimp  1901 AGAAGAACTG GACAATATGA TGGCAATCCC TTTGCAAACT TCAACTGTTA AAAATCATTC TGAAAATATC CTCCTGGAAA ATATCAACCA C----ACTTA
Rhesus 1842 AGAAGAATTA GATAATATGA TGACAATCAG TTCGCAAACT TCAACTGTTA AAAATCATTC TGAACCTATC CTACTGGAAG ATATCAGCCA C----ACTTA
Cow    1515 AGAAAAAATG GACAATATGA TGACAATCTC TTTGCAAACT TCAATTGTTA AAATTATTTT TGAACC-ATC CTGCTGGAAA AGAT----CA ACTGAACTCA
Dog    1586 AGAAAAATTG GACAACATGA TGACGATCCC TTTGTAAACT TCAATTGTc- AAAATCATCC TTGAACCATC CTACTGGAAA AGCTCAACCA A----ACTCA
            ***  ** *  ** **  **  ** * *     *  * ***** *    **     ** *  *   *  *   * * ** ******  *       **      **   

alignment
position    2201...... 2211...... 2221...... 2231...... 2241...... 2251...... 2261...... 2271...... 2281...... 2291......
Mouse  1603 CAAACAATTA TGTGTGTGAC AATCAATAAA CCTTTGACTG TGGAAATAAA GCTACTGAAT TACC---AAT AGCCAGAGAA CACTGTCTTC CAATAACAAA
Rat    1577 CAAACAATTA TGTGTGTGAC AATCAATACA CCTTTGACTA TGAAAATAAA ACTACCGAAT TACC---AAT AGCCAGAGAA CAGTGTCTTC CAATAACAAA
Human  1932 CAAACATTTA TTAGTATTAC CATAAATTAA TG-TTGACTG TGGCAATAAA AGTGCCATAA GAATTGTAAT AGCCAGGAAA ----ATCTTC TAAACAATTT
Chimp  1997 CAAACATTTA TTAGTATTAC CATAAATTAA TG-TTGACTG TGGCAATAAA AGTGCCATAA GAATTGTAAT AGCCAGGAAA ----ATCTTC TAAACAATTT
Rhesus 1938 CAAACATTTA TTATTATTAC CATAAATTAA TG-TTGACTG TGGCAATAAA AATGCAGTAA GAATTTTAAT AGCCAGGAAA ----ATATTC TAAATTATTT
Cow    1610 CAAACATTTA TTAGCATTAC AATAAATAAA CA-TTGATTA TGGCAATAAA AATGCCATAA GAATTTTAAG AGCTAGAAAA AACAATCTTC TAAATAAGGA
Dog    1681 AAAACATTCA CTAGCCTTAC AATAAACAAA CA-TTGACTA TGGTAATAAA AATGTCACAA GAATTTTAAT AGCTAATAAA AACAATCTTC TAAATAAAAA
             ***** * *       * **  **  *   *    **** *  **  ******   *     *   *     **  *** *   **      * ***  **        
```
